# Genome-wide DNA methylation landscape and its association with the transcriptome reprogramming in potato in response to *Phytophthora infestans* infection

**DOI:** 10.1093/hr/uhaf297

**Published:** 2025-11-04

**Authors:** Peng Tian, Jie Zheng, Bianbian Wang, Wenjing Jiao, Jianan Sang, Zhiyuan Ma, Pengcheng Han, Huimin Zhang, Yin Song, Yuling Meng, Weixing Shan

**Affiliations:** State Key Laboratory for Crop Stress Resistance and High-Efficiency Production, and College of Agronomy, Northwest A&F University, Yangling, Shaanxi 712100, China; Guangdong Provincial Key Laboratory for Plant Epigenetics, College of Life Sciences and Oceanography, Shenzhen University, Shenzhen, Guangdong 518060, China; State Key Laboratory for Crop Stress Resistance and High-Efficiency Production, and College of Agronomy, Northwest A&F University, Yangling, Shaanxi 712100, China; State Key Laboratory for Crop Stress Resistance and High-Efficiency Production, and College of Agronomy, Northwest A&F University, Yangling, Shaanxi 712100, China; State Key Laboratory for Crop Stress Resistance and High-Efficiency Production, and College of Agronomy, Northwest A&F University, Yangling, Shaanxi 712100, China; State Key Laboratory for Crop Stress Resistance and High-Efficiency Production, and College of Agronomy, Northwest A&F University, Yangling, Shaanxi 712100, China; State Key Laboratory for Crop Stress Resistance and High-Efficiency Production, and College of Agronomy, Northwest A&F University, Yangling, Shaanxi 712100, China; State Key Laboratory for Crop Stress Resistance and High-Efficiency Production, and College of Agronomy, Northwest A&F University, Yangling, Shaanxi 712100, China; State Key Laboratory for Crop Stress Resistance and High-Efficiency Production, and College of Agronomy, Northwest A&F University, Yangling, Shaanxi 712100, China; State Key Laboratory for Crop Stress Resistance and High-Efficiency Production, and College of Agronomy, Northwest A&F University, Yangling, Shaanxi 712100, China; State Key Laboratory for Crop Stress Resistance and High-Efficiency Production, and College of Agronomy, Northwest A&F University, Yangling, Shaanxi 712100, China; State Key Laboratory for Crop Stress Resistance and High-Efficiency Production, and College of Agronomy, Northwest A&F University, Yangling, Shaanxi 712100, China

## Abstract

Late blight, caused by the oomycete *Phytophthora infestans*, is one of the most destructive diseases affecting potato production globally. However, the function of DNA methylation (DNAm) and its association with simultaneous alteration in gene expression in potato’s response to *P. infestans* infection remain largely unknown*.* Here, we conducted whole-genome bisulfite sequencing and RNA sequencing on potato cultivar Qingshu No.9 inoculated with *P. infestans*. Significantly, we identified 18 119 differentially expressed genes (DEGs) across at least one of the four post-inoculation time points. A few pathogenesis-related (PR) genes involved in salicylic acid, ethylene signaling, and DNAm regulation exhibited activation at early infection stages, although they were predominantly downregulated after the onset of necrosis in plants. Hypomethylation changes at 12 h post-inoculation (hpi) were followed by hypermethylation at 24 hpi, with CHH methylation being the primary factor influencing the DNAm pattern. Differentially methylated regions (DMRs) showed significant enrichment at DEGs. Specially, DNAm variations could be associated with subsequent transcriptional changes. This is exemplified by 24 h-hyper-CHG methylation at the gene body that correlates with expression downregulation at 48 hpi, including genes involved in chromatin remodeling pathways. Furthermore, we observed a significant enrichment of hypomethylation changes at the exon of *NB-LRR* genes, which ultimately resulted in their downregulation. In summary, we have elucidated the DNAm pattern of potato in response to infection by *P. infestans*, and identified the involvement of epigenetic mechanisms in the reprogramming of the transcriptome, which ultimately contributed to the suppression of immunity and the development of potato late blight.

## Introduction

In the environment, plants are persistently exposed to a plethora of pathogens, including viruses, bacteria, fungi, and oomycetes. Such biotic stress affects plants at multiple levels, including growth, development, yield, and adaptability. To combat these pathogens, plants have evolved a sophisticated array of defense mechanisms, including the formation of the physical barriers on the cell surface and the synthesis of specialized metabolites that can be toxic to pathogens. Of particular importance is the active plant immune system, which includes pathogen-associated molecular pattern (PAMP)-triggered immunity (PTI) and effector-triggered immunity (ETI) [1, 2]. In PTI and ETI, plants employ cell surface-localized pattern-recognition receptors (PRRs) and intracellular nucleotide-binding domain leucine-rich repeat containing receptors (NB-LRRs), respectively, to recognize these pathogens and induce intricate downstream immune responses. These responses include the activation of mitogen-activated protein kinase (MAPK) cascades, reactive oxygen species (ROS) generation, and hormone signaling transduction [3]. Especially, the role of salicylic acid (SA), jasmonic acid (JA), and ethylene (ET) signaling pathways in pathogen defense responses is well established, with SA pathway involved in defense against biotrophic pathogens while JA/ET pathway involved in defense against necrotrophic pathogens [4–6]. Importantly, the activation of these immune responses is contingent upon extensive transcriptional reprogramming, which is tightly regulated by dynamic epigenetic modifications on the chromatin [7–9]. It is noteworthy that DNA methylation (DNAm), one of the most significant epigenetic mechanisms in eukaryotes, has also been demonstrated to be a crucial factor in the regulation of plant immunity, in addition to its pivotal roles in plant development and adaptation to environmental stresses [10, 11].

In higher plants, DNAm predominantly occurs at the fifth carbon of cytosine, which is referred as 5-methylcytosine (5mC). Depending on the sequence context, DNAm can be identified at the symmetric CG, CHG, and nonsymmetric CHH (H = A, C, T) sites. The maintenance of methylation at these sites relies on the activity of different DNA methyltransferases. Generally, MET1 is mainly responsible for the DNAm maintaining at CG sites. The enzyme CMT3 is mainly responsible for the maintenance of methylation at CHG sites. The CHH sites are primarily maintained by DRM2 and CMT2. Additionally, CMT2 is also responsible for a minor proportion of methylation at CHG [10, 11]. Besides, the RNA-directed DNA methylation (RdDM) pathway contributes to the *de novo* methylation that is mediated by small RNAs [10, 11]. Conversely, a family of bifunctional 5mC DNA glycosylases initiates active DNA demethylation [10, 11]. Thus, DNAm is regulated by a range of enzymes involved in various cellular processes, and exhibits variation during the response of plants to diverse stresses, including pathogen infection [7, 12].

Potato (*Solanum tuberosum* L.) is one of the most important food and vegetable crops that has been cultivated for >8000 years. It is the fourth most widely produced crop globally, after maize, wheat, and rice (https://cipotato.org/). Thus, the production of potatoes is of great significance to ensuring global food security. However, numerous diseases are a significant threat to the yield and storage of potatoes each year. One of the most damaging diseases affecting potatoes is late blight, caused by the oomycete pathogen *Phytophthora infestans*, which leads to economic losses of up to USD 10 billion annually [13]. As a hemibiotrophic pathogen, the infection process of *P. infestans* encompasses both an early biotrophic and a late necrotrophic stage. During the biotrophic stage, the pathogen obtains essential nutrients from living host cells. Once the biotrophic infection is well established, the pathogen shifts toward a necrotrophic lifestyle, obtaining nutrients from dead plant tissues. Subsequently, reproductive sporangiophores and sporangia will develop on the abaxial leaf surface [14]. The host’s response to the infection of *P. infestans* has yet to be fully characterized. Over the past few decades, a series of studies have been performed to elucidate the transcriptional reprogramming of potato to *P. infestans* infection [15–19]. These studies have demonstrated that potato’s transcription is altered during this process, with changes observed in the expression of genes involved in defense responses, hormone signaling, and metabolic pathways, among others. However, as one of the most crucial epigenetic mechanisms with close ties to transcriptional regulation, the manner in which DNAm responds and correlates with the defense response in this process remains largely unknown. To better understand the immune regulation in potato and aid in disease control and yield preservation, it is crucial to elucidate the role of DNAm in the plant’s response to *P. infestans* infection.

Qingshu No.9 (QS9) is one of the most propagated potato cultivars in China, that with high yield and excellent field performance against late blight for many years [20]. However, it does not display full immunity to *P. infestans* in the field. The disease incidence is significantly delayed, largely as a result of limited lesion expansion and development [17]. Thus, an in-depth analysis of the molecular mechanism underlying QS9’s response to *P. infestans* infection is required for its proper utilization and breeding. In this study, we aimed to elucidate the DNAm alterations in QS9 in response to *P. infestans* at the early stages of infection, and its associations with the transcriptome changes in this process. The transcriptome reprogramming of genes in different pathways was firstly characterized, with a particular focus on those related to defense response. The DNAm dynamic landscape was depicted at the genome-wide scale and at specific genes. Our findings demonstrate a significant association between DNAm alterations and transcriptional changes, including those related to disease resistance genes. In particular, we emphasize the positional and hysteretic effects of DNAm on gene expression. We propose that the suppression of the defense response in potato is accompanied by the downregulation of the photosynthesis system, the SA and JA/ET signaling pathways, disease resistance genes, and epigenetic regulation including but not limited to DNAm.

## Results

### Transcriptome reprogramming in potato in response to *P. infestans* infection

**Figure 1:**
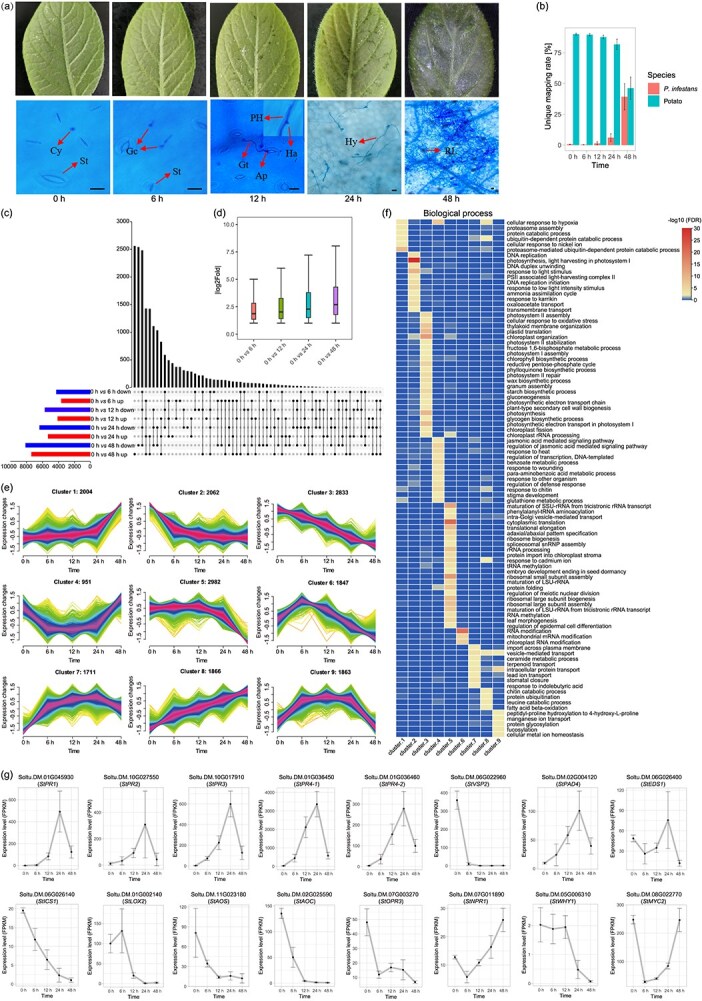
Gene expression changes in potato in response to *P. infestans* infection. (a) Appearance and microscopic observations of QS9 leaves inoculated with *P. infestans* zoospores at various time points. The top panel shows the appearance of potato leaves, the bottom panel shows the microscopic images at different stage. Ap, appressorium; Cy, cyst; Gc, germinated cysts; Gt, germ tube; Ha, haustoria; Hy, hypha; PH, primary hypha; RL, restricted lesions; St, stomata. The bar in these micrographs is 10 μm in length. (b) Mapping rate of RNA-seq reads to potato or *P. infestans* genome in different samples. (c) Number of differentially expressed genes in potato after infection. UpSet plots showing unique or shared up- or downregulated genes at different infection stages. ‘Up’ indicates upregulated expression as compared with 0 h, while ‘Down’ indicates downregulated expression as compared with 0 h. (d) Boxplot shows the absolute fold changes of DEGs at different stages. (e) Temporal dynamic pattern of DEGs. The red lines mean gene expression with higher membership values, followed by the blue, green, and yellow ones. The numbers above each figure show the number of genes in each cluster. (f) Heatmap of enriched GO terms for DEGs in different clusters. The heatmap values are transformed with -log10 (FDR), where FDR is the adjust *P*-value of GO enrichment analysis. Only GO term with -log10 (FDR) <2 in at least one cluster is exhibited. (g) Expression pattern of genes in SA and JA/ET signaling pathways.

To analyze the genome-wide DNAm and transcriptional response of potato to the infection by virulent *P. infestans,* leaves were collected at five time points, including 6, 12, 24, and 48 h post-inoculation (hpi), with noninfected leaves (0 hpi) serving as a control (Fig. 1a). At the initial time point of 6 hpi, no disease symptoms were observed. At 12 hpi, weak water-soaked lesions emerged on the leaves, subsequently becoming more pronounced at 24 hpi, accompanied by the appearance of a small number of white hyphae on the leaf surface. At 48 hpi, necrotic lesions were observed on the leaves. In accordance with the microscopic observations (Fig. 1a), germinated cysts were observed at 6 hpi. The germ tubes, appressoria, and primary hyphae were observed at 12 hpi. Then, haustoria and hyphae were observed growing between cells at 24 hpi. At 48 hpi, extensive mycelial growth was found within the leaves. These phenotypes suggest a typical compatible interaction between potato and the *P. infestans* isolate.

Following the inoculation of *P. infestans*, high-throughput RNA sequencing was performed to analyze the transcriptional response of potato to *P. infestans* infection, using material collected at the aforementioned five time points (Table S1). As expected, the unique mapping rates of the various RNA-seq samples to the potato reference genome declined with the prolongation of infection, while those to the *P. infestans* genome increased. This increase was particularly rapid, reaching an average of 39.25% at 48 hpi (Fig. 1b), suggesting significant proliferation of *P. infestans* at the onset of necrosis. The expression levels of genes in the three samples taken at the same time exhibited a high degree of correlation, with Pearson’s correlation coefficients ranging from 0.831 to 0.998 (Fig. S1a). Besides, gene clustering and principal component analysis (PCA) indicated that these biological replicates exhibit a high degree of replicability in gene expression profiles (Fig. S1b and c). Furthermore, quantitative real-time polymerase chain reaction (qRT-PCR) analysis of eight genes associated with the plant–pathogen interaction, including five RXLR effector genes and three defense response genes, confirmed consistent expression patterns between qRT-PCR and RNA-seq (Fig. S2). In total, 18 119 differentially expressed genes (DEGs), representing ~55% of the total genes, were identified in at least at one of the four post-inoculation time points. The number of downregulated genes exceeded that of upregulated genes at each time point, with the number of DEGs increasing with the progression of infection (Fig. 1c). Additionally, the fold changes of these DEGs also demonstrated a trend of increasing with the progression of infection (Fig. 1d). These findings indicate that as the infection progresses, the transcriptome reprogramming in plants becomes more abundant and pronounced.

A clustering analysis using Mfuzz [21] divided the 18 119 DEGs into nine clusters with distinct expression patterns (Fig. 1e). The Gene Ontology (GO) enrichment analysis showed that genes within distinct clusters exhibited a preference enrichment in disparate biological processes (Fig. 1f, Data S1), cellular components, and molecular functions (Fig. S3). For instance, genes associated with the chloroplast, photosystem, and photosynthesis were found to be enriched in Cluster-2 and Cluster-3, which demonstrated immediate downregulation following infection. Genes in Cluster-8, which exhibited continuous upregulation following infection, were found to be enriched in the processes of ‘chitin catabolic process’, ‘fatty acid beta-oxidation’, and ‘vesicle-mediated transport’. Interestingly, this upregulated ‘Chitin catabolic process’ involved seven *ChiC* genes (Class V chitinase, Fig. S4), which could be induced by plant stress-related hormones ABA, JA, and by the stress resulting from the elicitor flagellin, NaCl, osmosis [22]. And *ChiC* genes could also be induced by *phytophthora* pathogen analyzed here, suggesting their critical role in various stress-response processes. Notably, genes associated with the ‘jasmonic acid-mediated signaling pathway’, ‘regulation of defense response’, ‘response to chitin’, and ‘response to other organism’, that closely linked to defense responses, were enriched in Cluster-4. These genes were initially downregulated at 6 hpi and subsequently upregulated at 48 hpi. Genes in Cluster-6 exhibited rapid upregulation at 6 and 12 hpi, followed by downregulation after 24 hpi. These genes were significantly enriched in the process of ‘RNA modification’, including mitochondrial and chloroplast RNA modification. Especially, 141 genes in Cluster-6 were identified as the members of pentatricopeptide repeat (PPR) family (Fig. S5). These genes play a crucial role in RNA processing in mitochondria and chloroplasts, and have been shown to be involved in plant immunity by regulating ROS production [23, 24].

The expression changes of these orthologous genes in the SA and JA/ET signaling pathways were analyzed (Fig. 1g). The expression of *StPR1* and *StPR2*, which are related to SA, was significantly elevated at 12 hpi, reaching a peak at 24 hpi, and then decreased at 48 hpi. Similarly, *StPAD4* and *StEDS1*, which are responsible for SA signaling, exhibited an initial upregulation followed by a downregulation trend at 48 hpi. However, *StICS1* and *StICS2* (Fig. S6), the regulator in the pathway of SA synthesis, was observed to undergo a downregulation following the onset of infection. The expression of *StNPR1*, the key regulator in the SA pathway [25], was initially downregulated at 6 hpi but subsequently upregulated. *StWHY1*, a transcription factor (TF) in the SA pathway that is independent of *StNPR1*, did not undergo significant alteration during the initial stages of infection but was ultimately found to be downregulated. Additionally, *StPR3* and *StPR4* of the ET pathway exhibited an initial upregulation, followed by a downregulation. However, the JA-responsive marker gene *StVSP2* (Vegetative storage protein 2) was rapidly downregulated following infection. *StPDF1.2*, which is involved in both the JA and ET pathways, was silenced during this process. Similarly, *StLOX2*, *StAOS*, *StAOC*, and *StOPR3*, key components in the JA biosynthetic pathway, exhibited downregulation following infection. The expression of *MYC2*, the master regulator of JA signaling, was initially reduced but subsequently recovered. These findings indicate that only a subset of the SA and ET pathways was activated following infection and subsequently repressed at the end of the process, whereas the JA signaling pathway was predominantly repressed during the initial stages of infection.

### Differential modulation of DNA methylation regulators in the early infection stages

**Figure 2:**
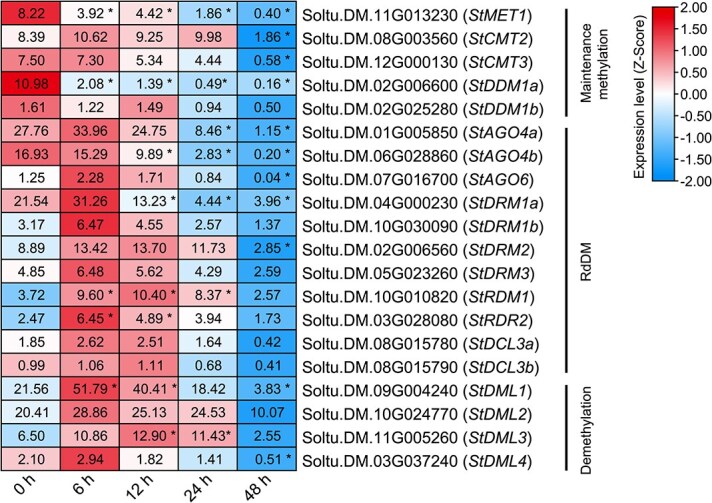
Expression pattern of DNA methylation regulators in potato in response to *P. infestans* infection. The heatmap is drawn based on *Z*-Score normalized expression values of each gene, with the original expression values (FPKM) maintained in these squares. The ‘*’ indicates the significant differential expression of the genes compared to the noninfected control at 0 hpi.

Before the revelation of the DNAm pattern, an initial analysis was conducted on the expression changes of genes associated with DNAm regulation (Fig. 2), including DNA methyltransferases, demethylase, and genes within the RdDM pathway [10]. The results indicate that a considerable number of these genes were expressed in a differential manner. *StMET1*, responsible for maintaining DNAm at CG sites [26], was found to be downregulated immediately following the infection. Similarly, *StDDM1a*, encoding a chromatin-remodeling ATPase involved in cytosine methylation in both CG and non-CG contexts [27], was also found to be downregulated following infection. In contrast, the four demethylase genes showed a tendency toward upregulation during the early stages of infection, with *StDML1* and *StDML3* displaying significant increases. Genes involved in the RdDM pathway also exhibited different expression pattern during the early stages of infection. For instance, *StRDM1*, a key component that forms the DDR complex with DMS3 and indispensable for PolV transcription [28], exhibited a rapid upregulation between 6 and 24 hpi. *StRDR2*, responsible for the synthesis of 24-nt heterochromatin siRNA [29], was also found to be upregulated at 6 hpi and 12 hpi. Differently, *StAGO4b*, responsible for the loading of 24-nt siRNA [30], and *StDRM1*, the methyltransferase involved in the *de novo* DNAm and maintenance at CHH sites [31], were downregulated after 12 hpi. Notably, nearly all the three groups of genes were significantly downregulated at 48 hpi, indicating that active DNAm regulation may be suppressed during the transition to necrosis in plants.

### Global DNA hypomethylation followed with hypermethylation changes in potato in response to *P. infestans* infection

**Figure 3:**
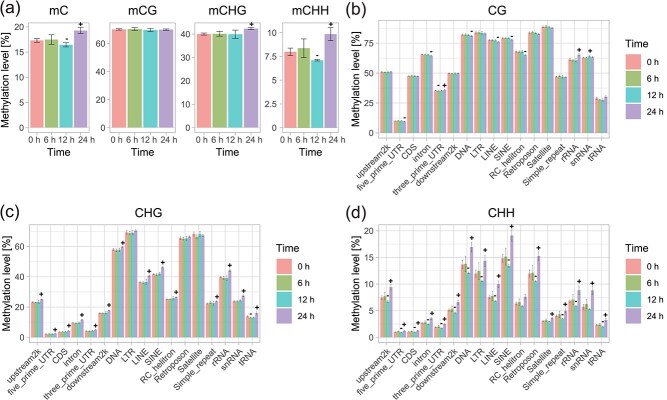
Global DNA methylation level changes in potato in response to *P. infestans* infection. (a) Global DNAm levels (mean ± SD) of different sequence contexts at different infection stages. mC includes all the three contexts. (b–d) Global methylation changes in CG (b), CHG (c), and CHH (d) contexts across different genomic regions during early infection stages. ‘+’ above the bar indicates a significant higher DNAm level of this time point than that of 0 h. ‘-’ above the bar indicates a significant lower DNAm level of this time point than that of 0 h. Significance was determined using a two-sample Student’s *t*-test with a threshold of *P* < 0.05.

To investigate the active DNAm regulation of potato in the early infection stages before necrosis, whole-genome bisulfite-seq was performed using the samples at 0, 6, 12, and 24 hpi (Table S2). In total, 3 684 547 636 reads, amounting to 552.7 Gb of data, were obtained, with the bisulfite conversion rates ranging from 99.58% to 99.70%. The average mapping rates of whole-genome bisulfite sequencing (WGBS) reads to the potato reference genome slightly declined from 79.09% at 0 hpi to 77.05% at 24 hpi. The average coverage depths ranged from 13.9 to 17.3, encompassing 80.8%–83.7% of the cytosines with a minimum of four reads in each library. The repeatability of the methylation levels among the three replicates from the same time points was high, with a Pearson’s correlation coefficient >0.98. These results demonstrate the high quality of our WGBS data. Based on the mapping results, the overall pattern of DNAm variation in the potato genome was revealed. The results showed that the overall DNAm (mC) level remained unaltered at 6 hpi, exhibited a slight decline at 12 hpi, and then followed by a notable increase at 24 hpi (Fig. 3a). Notably, the global DNAm changes were more consistent with that at the CHH sites. The methylation at CHG sites showed an increase at 24 hpi, whereas the global CG methylation remained stable across different time points (Fig. 3a).

As the heterogeneity of DNAm distribution around the genome, we analyzed the DNAm pattern of the three types in different genomic regions. In contrast to the global pattern, the changes in CG methylation were slight but diverse in different regions and at different stages (Fig. 3b). CHG methylation was predominantly upregulated at 24 hpi in the majority of regions, with exception of the highly methylated LTR, retroposon, and satellite regions (Fig. 3c). Obviously, the methylation alterations at CHH sites were largely consistent with the global pattern across different genomic regions (Fig. 3d), exhibiting a downregulation at 12 hpi and an upregulation at 24 hpi. These results indicate that the patterns of DNAm in different contexts are not consistent throughout the infection process. However, changes in CHH methylation were more pronounced and contributed to the global methylation pattern.

### DNA methylation variations approach gene regions from transposon elements during the infection process

**Figure 4:**
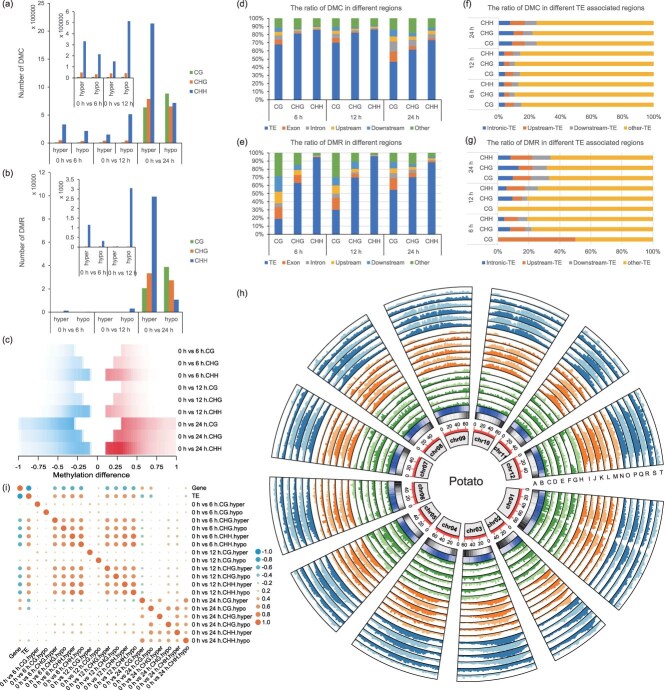
DNA methylation changes in potato at different *P. infestans* infection stages and their distribution patterns across the genome. (a) Number of DMCs at different stages. (b) Number of DMRs at different stages. (c) Intensity of DNAm changes of different contexts at different stages. ‘The shades of the color represent the density of DMCs at this scale. (d) Ratios of DMCs in different genomic regions. (e) Ratios of DMRs in different genomic regions. (f) Ratios of DMCs in different TE-associated regions. (g) Ratios of DMRs in different TE-associated regions. (h) Distribution densities of DMCs of different types, gene, and TE along the chromosomes in potato. The tracks A–T represent different genomic features, including Gene (A), TE (B), 6 h-CG-hyper (C), 6 h-CG-hypo (D), 12 h-CG-hyper (E), 12 h-CG-hypo (F), 24 h-CG-hyper (G), 24 h-CG-hypo (H), 6 h-CHG-hyper (I), 6 h-CHG-hypo (J), 12 h-CHG-hyper (K), 12 h-CHG-hypo (L), 24 h-CHG-hyper (M), 24 h-CHG-hypo (N), 6 h-CHH-hyper (O), 6 h-CHH-hypo (P), 12 h-CHH-hyper (Q), 12 h-CHH-hypo (R), 24 h-CHH-hyper (S), 24 h-CHH-hypo (T). Gene and TE densities are shown as heatmaps, while DMC densities are showed as bar plots. (i) Correlations among the distribution densities of DMCs of different types, gene, and TE. The distribution densities of DMC, gene, and TE along the genome were analyzed using a sliding window approach (100-kb window, 50-kb step). The correlation was calculated using Pearson’s correlation coefficient.

The global DNAm pattern suggests the difference of DNAm changes of the three contexts at different genomic regions. Furthermore, we analyzed these differentially methylated cytosines (DMCs) and differentially methylated regions (DMRs) in potato genome based on the comparison of DNAm levels of cytosines or regions between each after infection stage with that of noninfected. The results suggest that the numbers of CG- and CHG-DMCs were sparsely found at the two early infection stages. At 6 hpi, the number of hyper-CHH-DMCs (331 749) was 1.5 times more than that of hypo (213 993). Correspondingly, the number of hyper-CHH-DMRs (1151) was about three times more than that of hypo-CHH-DMRs (315), suggesting slight hyper-CHH methylation at 6 hpi. At 12 hpi, hypo-CHH-DMC (514 939) was significantly higher than hyper-CHH-DMC (149 441), and the number of CHH-hypo-DMR (3050) was dominant, with only a few CHH-hyper-DMR (32), which is consistent with global CHH-hypomethylation pattern at this stage (Fig. 4a and b). At 24 hpi, not only the numbers of DMCs and DMRs of the three types (Fig. 4a and b), but also the strength of methylation changes (absolute methylation level difference between infected and noninfected) at 24 hpi were larger than that of the two previous stages (Fig. 4c), indicating dramatic methylation changes around the whole genome at 24 hpi. Besides, the number of hyper-CHH-DMC (2 136 257) was nearly three times more than that of hypo-CHH-DMC (714 286), and the number of hyper-CHH-DMRs (99 189) was nine times more than that of hypo-CHH-DMRs (10 664) (Fig. 4b).

The distribution pattern of DMC and DMR in various genomic regions showed that most of the methylation changes occurred in the transposon elements (TE) region, and the proportion of CHH methylation changes in the TE region was the largest, followed with CHG and CG. On the contrary, the proportions of CG methylation changes in gene body (including both exon and intron) and its flanking regions were higher than that of CHG and CHH. As compared with the two previous time points, we noticed that the proportions of methylation changes in genes and their flanking regions were increased at 24 hpi (Fig. 4d and e). Besides, the proportions of methylation changes in TEs were also significantly increased in those associated with genes, including intronic TEs or TEs overlapping the 2-kb gene-flanking regions (Fig. 4f and g). These results indicate that the methylation changes at 24 hpi tend to approach gene regions.

The DNAm pattern at 24 hpi suggests that genomic feature is not the only cause that determine the distribution of DNAm variation. To further characterize the DNAm changes and the difference among different cytosine contexts, we analyzed the correlation among the distribution densities of DMCs of different types, and at different time points, with the distribution densities of gene and TE (Fig. 4h and i). Due to the small number of CG-DMCs at 6 and 12 hpi, there was no significant correlation of them with the other DMCs. The distributions of CHG-DMC and CHH-DMC at 6 and 12 hpi were negatively correlated with gene distribution (*r* = −0.615 to −0.491, *P* < 2.2e−16), but positively correlated with TE distribution (*r* = 0.505–0.631, *P* < 2.2e−16). And there were positive correlations (*r* = 0.562–0.841, *P* < 2.2e−16) among the distribution of CHG and CHH-DMCs at 6 and 12 hpi, suggesting that these non-CG methylation changes at the two early stages were more consistent, and mainly occurred in TE regions. At 24 hpi, the distribution of CG methylation was positively correlated (*r* = 0.394–0.422, *P* < 2.2e−16) with gene but negatively correlated (*r* = −0.462 to −0.420, *P* < 2.2e−16) with TE. However, this distribution preference of CHG- and CHH-DMCs to TEs was altered at 24 hpi, with the correlation coefficients to TEs were largely reduced (*r* = −0.223 to 0.054, Fig. 4i). Moreover, the hypo-CHH DMC even showed positive correlation (*r* = 0.178, *P* < 2.2e−16) with gene but negatively correlated (*r* = −0.223, *P* < 2.2e−16) with TE distribution at 24 hpi. In addition, at 24 hpi, we also noticed that the distribution of CG-hyper-DMC was positively correlated with CHG-hyper-DMC (*r* = 0.492, *P* < 2.2e−16) and CHH-hypo-DMC (*r* = 0.688, *P* < 2.2e−16) distribution. In contrast, the distribution of CG-hypo-DMC was positively correlated with CHG-hypo-DMC (*r* = 0.638, *P* < 2.2e−16) and CHH-hyper-DMC (*r* = 0.628, *P* < 2.2e−16). This might indicate the coregulation among different types of DNAm, especially the direction of DNAm changes tend to be consistent at the symmetric sites (CG, CHG), but with inverse alteration at the nonsymmetric CHH sites. Moreover, previously we found that the distributions of hyper- and hypomethylation changes of the same context were positively correlated in tomato’s response to phosphate starvation stress, a potential strategy to maintain the stability of the epigenome [32]. Though we found positive correlation (*r* = 0.095–0.841, *P* ≤ 4.87e−16) between the distribution of hyper- and hypomethylation changes of the same context in this research, the correlations of CHG (*r* = 0.095) and CHH (*r* = 0.360) types at 24 hpi were obviously lower than that of the two previous stages (CHG, *r* = 0.562–0.60; CHH, *r* = 0.812–0.841), which might indicate the imbalance of DNAm changes when a plant is under severe pathogen stress.

### Positional and hysteretic effects of DNAm on gene expression

DNAm at gene promoter or body regions can regulate gene expression [10, 33–35]. Using a similar method as in previous work [36], we also found that non-CG DNAm in the gene body regions and methylation levels in the upstream region near the transcription start site (TSS) were negatively correlated with gene expression, moderately expressed genes had higher CG methylation levels than lowly and highly expressed genes, and CHH methylation positively correlated with gene expression in the upstream region (200–800 bp) of the TSS (Fig. S7). However, when considering the transition from noninfected to infected status, it remains undetermined whether DNAm changes correlate with alterations in gene expression.

**Figure 5:**
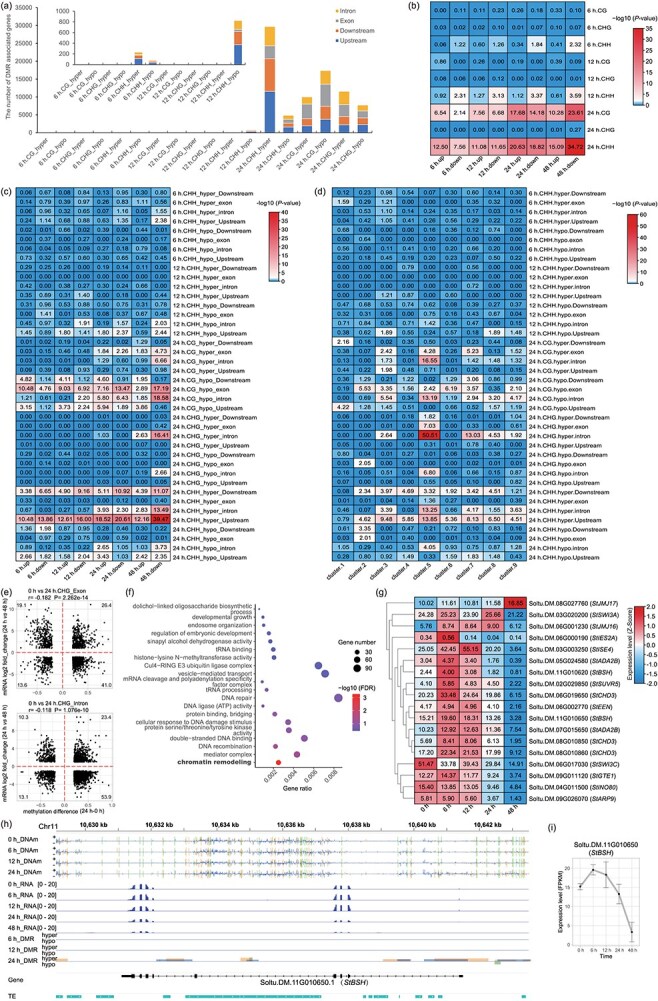
The association of DNA methylation change with gene expression alteration in potato after *P. infestans* infection. (a) Number of different types of DMR-associated genes. (b) Association of DMRs of different contexts with DEGs at different time points. (c) Association of DMRs of different contexts, different time points, and different regions with DEGs at different time points. (d) Association of DMRs of different contexts, different time points, and at different regions with DEGs in different clusters. In figure b–d, the value in the square represents the -log_10_ transformed *P*-value that used to estimate the enrichment of different types of DMRs around different types of DEGs by Fisher’s exact test. (e) DNAm changes of 24 h-CHG-DMR at gene body, including exon and intron, tend to be negatively correlated with delayed gene expression alterations at 48 hpi. Numbers in the four corners show the percentage (%) of points in each area. (f) The GO term enrichment analysis of 24 h-CHG-DMR-associated genes. (g) The expression profile of chromatin remodeling genes with 24 h-CHG-hyper DMRs at their body. (h) Genome browser screenshot of the chromatin remodeling gene *StBSH* locus, showing the methylation levels, expression levels, and DMRs at different stages. Different cytosine contexts or different DMRs are coded by colors (green for CG, orange for CHG, and blue for CHH); the expression level is normalized by reads coverage density as RPM in the square. DMRs are shown as boxes with the width and height to show the region and difference level; the gene structures are shown with black boxes as coding exons, narrow black boxes as untranslated regions (UTRs), lines as introns, and the arrows indicating transcription directions. TEs are presented as cyan boxes with the arrows indicating their strand directions. (i) Expression pattern of the *StBSH* gene.

To explore this issue, the DMR-associated genes (DMG) were identified according to the locations of DMRs at genes, including DMRs that existed within the gene body, 2 kb upstream or downstream of the gene. Finally, we identified 11 CG-, 23 CHG-, 310 CHH-DMGs at 6 hpi; 6 CG-, 15 CHG-, 827 CHH-DMGs at 12 hpi; and 18 241 CG-, 13 611 CHG-, 21 968 CHH-DMGs at 24 hpi, respectively (Fig. 5a). CHH-DMRs at 6 and 12 hpi have few overlaps between each other, but co-occurred with a few CHH-DMRs at 24 hpi (Fig. S8a). Obviously, more overlaps of DMRs of different contexts were found at 24 hpi. Especially, co-occurrence of CG- and CHG-hypomethylation were identified at the exons of 767 genes, with CHH-hyper-DMRs frequently found at the flanking regions of these genes (Fig. S8b). Besides, CHG-hyper methylation at exon or intron also accompanied with CHH-hyper-DMR at these regions (Fig. S8b). The results further confirmed the coregulation of DNAm of different contexts at gene regions as previously mentioned (Fig. 4i).

To assess whether these DMRs related to the gene expression changes were caused by *P. infestans* infection, we evaluated the overlapping enrichment of DMG of different types with DEG at different time points by Fisher’s exact test, which was to determine whether DMRs occurred significantly in these DEGs. The results showed that DMRs of different contexts at 6 h exhibited no enrichment at DEGs of the same time, while CHH-DMRs at 12 h, CG- and CHH-DMRs at 24 h showed significant enrichment (*P* < 0.01) at simultaneous DEGs (Table 1). Especially, CHH-DMR at 12 h, which were mostly hypomethylated, enriched mainly at downregulated genes. Notably, except for the sparse CG- and CHG-DMRs at 6 and 12 h and the CHG-DMRs at 24 h, DMRs were enriched around DEGs of subsequent time points, indicating a hysteresis effect between DNAm and transcriptional changes (Fig. 5b). Considering the regional effect of DNAm to gene expression (Fig. S7), we divided these DMRs according to their locations at genes (exon, intron, and flanking 2 kb) and variation trends (hyper or hypo), and assessed the enrichment of different DMR types in DEGs at various stages (Fig. 5c), or DEGs in diverse expression clusters (Fig. 5d). Different with the overall pattern, CHH-DMRs at different regions of 6 and 12 hpi showed no enrichment at DEGs of the same time or DEGs from any cluster. Only DMR of 6 h-CHH-hyper-upstream (CHH hyper-DMR identified at 6 hpi and located in the upstream of gene) and 12 h-CHH-hypo-upstream showed slight enrichment at subsequent downregulated genes. Notably, at 24 h, CG hypomethylation at the flanking region mainly enriched at upregulated genes, and CG-hypo-upstream also showed enrichment in Cluster-1 that was finally upregulated. CG-hypo at exon, CHH-hyper at flanking region showed significant association with DEGs of the same time point and the other time points, and DEGs from different clusters. For DEGs of the Cluster-4, we detected significant enrichment of 24 h-hyper-CHH DMRs at the flanking regions. Interestingly, 24 h-CHG-hyper-intron showed significant associations at downregulated genes at 48 hpi and DEGs in Cluster-5, which also showed downregulation at 48 hpi. These results suggest that DNAm changes at 24 hpi for the three contexts and within specific positions could be significantly associated with the simultaneous and subsequent gene expression changes, with obvious position and hysteresis effect of DNAm changes on gene expression.

**Table 1:** Enrichment of DMRs at DEGs found at different time points

DMR	DMG	6-h DEG overlap (7927)	Fisher’s *P*-value	12-h DEG overlap (9817)	Fisher’s *P*-value	24-h DEG overlap (11 684)	Fisher’s *P*-value	48-h DEG overlap (15 504)	Fisher’s *P-*value
6 h.CG	11	1	.952	3	.683	4	.588	5	.657
6 h.CHG	23	2	.985	4	.944	4	.984	8	.919
6 h.CHH	310	79	.301	108	.032	127	.026	169	.005
12 h.CG	6	2	.444	1	.88	1	.928	3	.6
12 h.CHG	15	2	.908	3	.87	1	.999	1	1
12 h.CHH	827	236	.002	298	5.83e−05	348	4.28e−05	442	.0001
24 h.CG	18 241	4609	1.05e−08	5775	2.35e−16	7036	4.45e−39	9227	1.49e−45
24 h.CHG	13 611	2868	1	3578	1	4526	1	6334	.958
24 h.CHH	21 968	5637	6.44e−22	6963	1.89e−26	8391	1.53e−48	11 081	7.50e−67

The number in the brackets indicate the total number of DEGs at this stage. Fisher’s exact test is used to analyze whether DMGs is significantly found in DEGs.

Thus, we further calculated and compared the correlation of DNAm changes with the expression fold changes of associated DEGs at the same time points. Unexpectedly, the results suggest that there was no significant correlation between the methylation changes and the expression fold changes of associated DEGs at the same time (Fig. S9). Even considering the positional effect, DNAm changes of DMRs at different regions also showed no significant correlation with the expression changes of associated genes (Fig. S10). Besides, considering the hysteresis effect between DNAm and gene expression, we calculated the correlation of 24-h DNAm changes with the expression fold changes of DEGs at 48 h. The results suggested that only CHG methylation changes at exon and intron showed weak negative correlation with the gene expression changes (Fig. 5e). And DEGs with hyper-CHG-DMRs at their body mainly involve in the processes such as ‘cytosol’, ‘chloroplast’, and ‘intracellular protein transport’ (Fig. S11). Generally, these results indicate that the alteration of DNAm level in a specific region does not definitely indicate gene silencing or activation. As a proof, we compared the methylation pattern of samples between 0 and 24 h at three types of genes, including upregulated, downregulated, and non-DEGs of the same time. The results indicate that the pattern of average methylation changes between the two stages were consistent among the three types of gene, that the average CG methylation level turned to be stable, while average CHG and CHH methylation were increased (Fig. S12). In addition, 24 h-DMRs, especially CHG-hypomethylation, could enrich at non-expressed genes (Table S3), further suggesting DNAm changes were not always related with gene expression changes.

To investigate whether DNAm changes preferentially occur at genes with specific function, we performed GO enrichment analysis of these DMGs at different time points and of different types. The results suggest that only 24 h-CHG-DMG was specifically enriched (*P*_adj_ = 5.16e−04) in ‘chromatin remodeling’ (Fig. 5f). Notably, CHG-DMRs were predominantly hypermethylated (78.2%) and located in the body (64.4%) of these chromatin remodeling genes. Furthermore, many of these CHG-hypermethylated chromatin remodeling genes exhibited a trend of downregulation at 48 hpi, involving several critical components of these chromatin remodelers like INO80 (*StINO80*, *StARP9*, *StEEN*, *StIES2A*), CHD (*StCHD3* homologs), and SWI/SNF (*StSWI3C*, *StBSH*) (Fig. 5g). For instance, BSH is a conserved subunit of plant SWI/SNF complexes [37]. At least four CHG-hyper DMRs were detected at the body of *StBSH* at 24 hpi. Besides, CHH-hyper DMRs also coexist with these CHG-hyper DMRs (Fig. 5h). Meanwhile, *StBSH* turned to be downregulated at 48 hpi (Fig. 5i), indicating the suppression of chromatin remodeling gene by non-CG methylation in this susceptible process. For genes in the SA and JA/ET signaling pathways, significant DNAm changes were also observed. Particularly, CHH hyper-DMRs were frequently observed at these genes with a trend of upregulation at 24 hpi (Fig. S13a–h), including the SA-related genes (*StPR1*, *StPR2*, *StPAD4*, *StEDS1, StNPR1*), ET pathway genes (*StPR3*, *StPR4*), and the JA regulator MYC2. Moreover, nearly all of these CHH-hyper DMRs are located in TEs within introns or inside flanking regions of these upregulated genes, indicating the hyper-CHH methylation may be associated with the maintaining of heterochromatin islands as previously reported [32, 38]. Additionally, DNAm changes could also be significant at these downregulated SA (*StICS1*)- or JA-related genes (*StLOX2*, *StAOS*, *StOPR3*), and mainly happened in the gene body (Fig. S13i–l). Interestingly, for *StVSP2* and *StAOC* that with ultralow DNAm at gene body (Fig. S13m and n), DNAm changes were rarely observed, suggesting other factor involving in the regulation of these genes. In all, these results highlight the positional and hysteretic effects of DNAm on gene expression in various processes including defense response and chromatin organization regulation.

### Hypo-DNAm changes are enriched at the exons of *NB-LRR* genes

**Figure 6:**
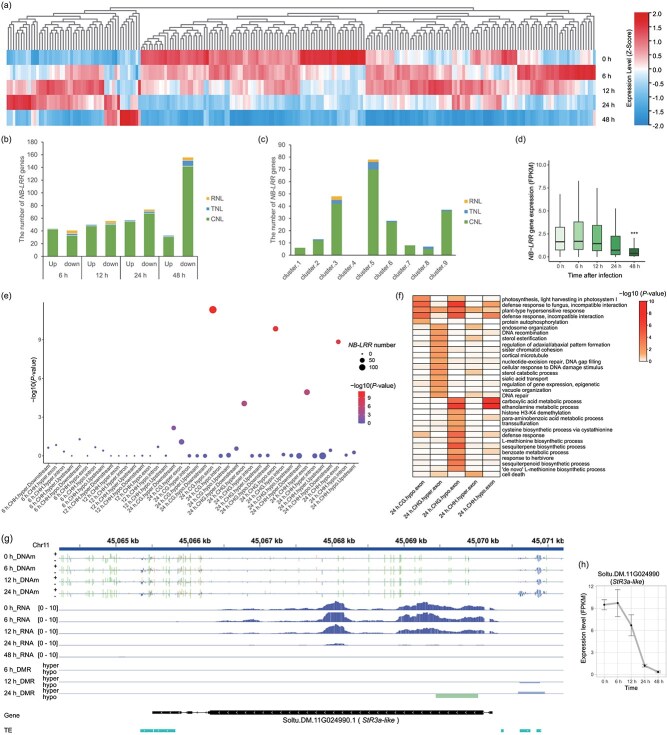
Expression and DNAm changes at the *NB-LRR* genes. (a) Heatmap shows the expression profile of *NB-LRRs* at different stages. (b) Numberof up- and downregulated *NB-LRR* genes after infection. (c) Number of *NB-LRR* genes in different clusters with distinct expression pattern. In figure b–c, the abbreviation TNL represents TIR-NBS-LRR, CNL represents CC-NBS-LRR, and RNL represents RPW8-NBS-LRR. (d) Global expression level of *NB-LRR* genes in potato after *P. infestans* infection. ‘***’ indicates significant difference (*P* < .001, two-sample Student’s *t*-test) between this stage with that of 0 h. (e) Enrichment of DMRs of different type and in different regions within *NB-LRR* genes. (f) GO biological processes of different DMR-associated genes. (g) Genome browser screenshot of the *StR3a-lik*e locus, showing the methylation levels, expression levels, and DMRs at different stages. The tracks are similar to that showed in Fig. 5h. (h) Expression pattern of the *StR3a-lik*e gene.

It is notable that *NB-LRR* genes play a significant role in detecting pathogens and the subsequent activation of ETI. However, a comprehensive understanding of the global association between DNAm and *NB-LRR* expression in the context of plant susceptibility remain elusive. A total of 466 *NB-LRR* genes were annotated in the potato genome, comprising 438 owing to *CNLs*, 23 to *TNLs*, and 5 to *RNLs* (Data S2). The transcriptome analysis revealed that the majority of *NB-LRRs* showed low expression levels, with 57.1%–87.6% of them obtaining a Fragments Per Kilobase of transcript per Million mapped reads (FPKM) <1 in each stage (Fig. S14). Among these genes, 225 *NB-LRR* genes showed differential expression during the infection process (Fig. 6a). A total of 93 *NB-LRR* genes were transiently upregulated during the infection process, only to be rapidly downregulated shortly thereafter (Fig. S15a). Only 9 genes exhibited significantly higher expression throughout the infection process than those observed in noninfected tissues (Fig. S15b). However, with the exception of 6 hpi (43 up vs 41 down), the number of downregulated *NB-LRRs* exceeded that of upregulated *NB-LRRs* at the other three time points. This trend was particularly evident at 48 hpi (33 up vs 156 down, Fig. 6b). All the *RNLs* were found to be downregulated (Fig. 6b). Consistently, the differentially expressed *NB-LRRs* were more prevalent in Cluster-5, adjacent to Cluster-3 and Cluster-9 (Fig. 6c). Furthermore, the total expression level of *NB-LRRs* was quantified throughout the infection process. This revealed a downregulation in the overall expression level of *NB-LRRs* with the progression of infection (Fig. 6d). Especially, 79 *NB-LRRs* showed the highest expression in the noninfected stage compared to other stages after infection, suggesting *NB-LRRs* were not always activated under pathogen stress.

The characteristics of DNAm surrounding *NB-LRR* genes were examined and revealed notable distinctions when compared to those of randomly selected genes (RSGs). Specifically, *NB-LRRs* show elevated CG methylation at the flanking region and TSS sites, while displaying reduced CG methylation at the gene body in proximity to the TES when compared to RSGs. In contrast, *NB-LRRs* exhibit reduced CHG methylation at a distance of 2 kb upstream of the TSS, while displaying elevated CHG methylation at the gene body and 2 kb downstream of the TES. Furthermore, *NB-LRRs* exhibit reduced CHH methylation in the vicinity of 200–800 bp upstream of the TSS, while displaying elevated CHH methylation at the gene body (Fig. S16). The RSGs exhibit a similar proportion of expression scales as the total gene, whereas *NB-LRRs* show a greater distribution at the scales <3 FPKM (Fig. S14). In light of the preceding results (Fig. S7), it can be postulated that the methylation pattern observed around *NB-LRRs* is consistent with their relatively low expression in potato.

To investigate the potential association between DNAm alternations and the *NB-LRR* genes, we assessed the enrichment of DMRs within *NB-LRR* gene family. The results indicated that 24 h-hypo-DMRs of all the three contexts at exon regions demonstrated notable enrichment at *NB-LRR* genes. Furthermore, 24 h-hyper-DMRs of CHG and CHH at exons were also enriched at *NB-LRR* genes, although to a lesser extent than these hypo-DMRs (Fig. 6e). The results of the GO enrichment analysis showed that these DMRs of the five types were enriched at the ‘plant-type hypersensitive response’, while hypo-DMRs of the three contexts demonstrated a greater enrichment at defense response-related process than hyper-DMRs (Fig. 6f). Besides, we observed that DMRs of different contexts could co-occur at the exons of the same *NB-LRR* genes, with a notable prevalence of hypo-DMRs of different contexts or hyper-DMRs of these non-CG types (Fig. S17). In addition, among the 767 genes exhibiting the co-occurrence of CG- and CHG-hypo-DMRs at their exons (Fig. S8b), a significant enrichment of them in the categories of ‘defense response’ and ‘plant-type hypersensitive response’ was also identified, which included 36 *NB-LRR* genes (Fig. S18). In conclusion, the result indicated a preference for hypo-methylation changes at the exon of *NB-LRR* genes. For instance, *R3a* is one of the most well-studied *R* gene in potato that confers race-specific resistance to *P. infestans* [39]. Previous research has confirmed the potential existence of *R3a-like* gene in QS9 based on its recognition of the effector gene *Avr3a^EM^* [40]. The ortholog of *R3a* is located at *Soltu.DM.11G024990* in the reference genome [41], although there is significant sequence divergence from the original *R3a* (Fig. S19). Our analysis showed that this *StR3a-like* gene also showed reduced expression during the infection process, with a relative higher expression level in the noninfected stage (Fig. 6g and h). A 24 h-hypo-CG-DMR was identified at the exon of the *StR3a-like* gene, along with a 24 h-hyper-CHH DMRs at its promoter (Fig. 6g). In accordance with the global pattern, the correlation between DNAm changes at the exon and the expression changes of these *NB-LRRs* was not statistically significant. Some DMRs were identified in *NB-LRRs* that were not expressed. For the 149 *NB-LRRs* with 24 h-hypo-CG-DMR, 24 showed upregulation and 28 exhibited downregulation. Additionally, 82 of these *NB-LRRs* showed low expression (FPKM < 1) at this stage, indicating a preference for hypo-CG methylation at low-expression genes. Previous reports indicated that 24 h-hypo-CG-DMR at exon were enriched at DEGs with a trend of downregulation in this process or at 48 hpi, including the Cluster-2, -3, -5, -6, -7, and-9 (Fig. 5d). However, 24 h-hypo-CHG-DMR and 24 h-hypo-CHH-DMR did not show any enrichment at DEGs of the same time (Fig. 5c). Nevertheless, they were enriched in cluster-2, which was downregulated immediately at 6 hpi (Fig. 5d). Taken together, these results indicated that DNAm changes were significantly found at the exons of *NB-LRRs*, particularly associated with their downregulation during the progression of *P. infestans* infection.

## Discussion

DNAm plays an important role in plant immunity [7, 8, 12]. Although our study focused solely on characterizing the transcriptome and DNAm changes in potato during the susceptible phase to *P. infestans*, susceptibility should not be equated with plant passivity and the loss of resistance. In fact, active host cooperation may be required for the pathogen to propagate and cause disease [42]. Therefore, understanding the mechanisms underlying the susceptible response is essential for comprehending plant–pathogen interactions and for informing agricultural practices.

WGBS has enabled the revelation of the dynamic changes of DNAm at the genome-scale that occur during a plant’s response to pathogens or other organisms. Especially, a reduction in methylation levels of plants has been frequently observed to be associated with an enhanced resistance response. For example, following infection with the obligate biotrophic fungus *Blumeria graminis* f. sp. *tritici*, the wheat diploid progenitor showed a notable decline in CHH methylation, accompanied by downregulation of *AGO4a* in the RdDM pathway [43]. Furthermore, CHH hypomethylation was observed to be enriched at the adjacent transposon elements of genes associated with the function of ‘response to stress’, including receptor kinase, peroxidase, and pathogenesis-related genes. Similarly, upon treatment with nematode-associated molecular patterns from different nematode species, or the bacterial PAMP flg22, global DNA hypomethylation was found in both rice and tomato, with the majority of changes occurring at CHH sites. Interestingly, the observed changes in DNAm were not correlated with gene expression at the same time point, but were correlated with delayed transcriptional activation of genes [44]. Furthermore, it was shown that the feeding of aphids leads to DNA hypomethylation at hundreds of loci in *Arabidopsis*, with methylation alterations predominantly occurring at transposable elements, which were associated with altered expression of numerous immune response genes [45]. However, hypomethylation is not a universal phenomenon in plants following pathogen infection. For instance, following infection with the blast fungus *Magnaporthe oryzae*, global DNAm levels in rice exhibited a slight increase, mainly at CHH sites and in association with transposable elements [46]. Fortunately, the DNAm response of several other plants to different *phytophthora* and oomycetes pathogen were also reported in recent studies, and suggested DNAm serving as potential defense mechanism [47–50]. Interestingly, distinct molecular responses of susceptible and tolerant plants to the same pathogen were revealed [47–49]. In soybean’s response to *Phytophthora sansomeana*, nonsignificant global DNAm changes were identified in both the susceptible (Williams 82) and resistant (Colfax) variety. Local methylation changes, specially an increase in CHH methylation around genes and TEs after inoculation, which occurred earlier in the susceptible line and later in the resistant line [48]. Besides, more TEs exhibited changes in their methylomes in the susceptible line compared to the resistant line. In addition, DMR-associated genes also showed different enrichment, with Colfax primarily showing involvement in metabolic process, defense response, plant and pathogen interaction, anion and nucleotide binding, and catalytic activity, while Williams 82 exhibited a significant association with photosynthesis [47], which is similar as we observed in potato’s susceptible process. Specifically, we observed an initial hypomethylation followed by hypermethylation at the early stages of infection before the plants exhibited necrosis. This pattern contrasts with the methylation changes reported in other plants infected with various pathogens [43, 45–47], indicating that the DNAm response pattern is diverse and may depend on the specific interactions between plants and pathogens, and the infection stages that were analyzed. Consistent with these previous studies, changes in methylation at CHH sites primarily shaped the variation pattern, highlighting the sensitivity of CHH sites to stress. Notably, CHH hypomethylation was particularly evident at 12 hpi and appeared to be a global response pattern, with a preference for TE regions (Figs 3 and 4). Although we found a relative enrichment of 12 h-CHH-DMRs around DEGs at the same time point (Fig. 5b), these DMGs showed less enrichment at defense response genes. This finding contrasts with several previous reports that CHH hypomethylation is associated with the activation of stress or immune response genes following pathogen or insect attack [43–45], suggesting different role or impaired effect of CHH hypomethylation to defense activation in this study. Possibly, the hypomethylation in potato was not strong and lasting enough in this quick susceptible process. Obviously, DNAm changes at 24 hpi tend to be dramatic and exhibited a distribution preference at genes as compared with the two earlier stages. Consistently, we observed significant enrichment of DMRs of CG and CHH types around DEGs at this time (Fig. 5b). Thus, our results suggest that DNAm is dynamically regulated and plays different roles at different stages after infection.

We highlighted the positional effect of DNAm changes with gene expression alterations. Especially, after considering the positional effect, the association of DMR to DEGs were much different. For example, 24 h-CG hypomethylation at the flanking region mainly enriched at upregulated genes or DEGs with a trend of upregulation at the 48 hpi (Cluster-1, Fig. 5c and d). In particular, CHG-DMRs showed no enrichment around DEGs when the position and methylation trend were not considered. However, 24 h-hyper-CHG-DMRs at intron showed significant enrichment at downregulated genes at 48hpi, or genes with a trend of downregulation (Cluster-5, -7, Fig. 5d). These findings also indicated that DNAm were not well coordinated on time with gene expression changes, with a hysteresis effect between them during this process. On one hand, we only found slight CHH changes at 6 hpi, while the expression of several DNAm regulators were altered at 6 hpi, including the upregulation of *StDML1*, *StRDR2*, *StRDM1* and the downregulation of *StMET1*, *StDDM1a* (Fig. 2). Although these genes may contribute to opposite alterations in DNAm level, these results indicate the expression changes of DNAm regulators do not lead to immediate DNAm changes. On the other hand, DNAm changes might also not lead to simultaneous gene expression changes, as supported by the enrichment of these DMRs around DEGs not only at the same time point, but also at DEGs of the subsequent time point (Fig. 5b and c). Additionally, a weak negative correlation was found between the 24-h gene body CHG methylation changes with the gene expression alterations at 48 hpi (Fig. 5e). Consistently, it has been reported that nematode-pattern-triggered CHH hypomethylation was correlated with a delayed transcriptional gene activation rather than gene expression changes at the same time point [44]. This suggests that the hysteresis effect between DNAm and gene expression was not a rare case, which might partially explain why DNAm changes at these DMRs were not significantly correlated with associated gene expression changes (Fig. 5e, Fig. S6).

The positional and hysteresis effect were also well exemplified in *NB-LRR* genes. Significant enrichment of DMRs, especially hypomethylated DMRs, was found in the exons of these disease resistance genes, which exhibited a trend of silencing at the end of the infection. We propose that hypomethylation changes in the exons may mark or predict the downregulation or low expression of these *NB-LRR* genes during this susceptible process. Notably, QS9 exhibited global downregulation of *NB-LRRs* and relatively high expression of a group of *NB-LRRs* in the noninfected state (Fig. 6a and d). To elucidate whether this expression pattern was specific in the cultivar QS9 or a property in the susceptible process, we analyzed several public transcriptomic datasets derived from potatoes inoculated with *P. infestans* (Table S4). This includes the susceptible process of living QS9 after *P. infestans* infection [17], the resistant or susceptible states of the primitive cultivated potato (*Solanum andigena*) inoculated with *P. infestans* strains with different virulence [16], and the resistant and susceptible states of different potato materials inoculated with the same strain [15]. Interestingly, the global expression pattern of *NB-LRRs* showed more significant downregulation in the susceptible process of QS9 living leaves (Fig. S20a), with 208 *NB-LRRs* obtaining the highest expression in the noninfected stage (Fig. S20d). During the resistant process of potato, the overall expression of *NB-LRR*s did not show obvious changes in two independent experiments (Fig. S20b and c). In contrast, during the susceptible process of Favorita, the *NB-LRRs* showed an upward trend (Fig. S20c and f), while the expression in *S. andigena* did not change significantly, with only a slight downregulation in the early susceptible stage (Fig. S20b and e). These results suggest that the expression patterns of *NB-LRRs* might be associated with the susceptibility status of the plants. Each potato cultivar possesses a group of *NB-LRRs* with a relative high expression in the noninfected stage, which is more prominent in QS9. The eventual manifestation of susceptibility in plants following pathogen infection suggests that the ETI pathway might be either inhibited or not activated. Possibly, the relatively high expression of *NB-LRRs* in QS9 in the absence of infection might enable more rapid recognition of pathogens, thereby facilitating a more expeditious immune response. Conversely, if the resistance genes fail to recognize certain virulent strains, leading to a susceptible phenotype, the downregulation of *NB-LRRs* may serve to reduce energy consumption, thereby enhancing the tolerance of QS9 to disease as previously reported [17].

CHG methylation is maintained by CMT3, and CHG methylation and suppressive histone H3K9me2 modification can reinforce each other through regulatory feedback loops [10], which might stand as reason for the suppressive role of CHG methylation at these downregulated genes. It is noteworthy that CHG hypermethylation is enriched at genes in chromatin remodeling pathway, and may be associated with the suppression of these genes in the transition to necrosis stage (Fig. 5f–i). Closely related to other epigenetic modifications, chromatin remodeling refers to the dynamic structural changes in chromatin that allow access to DNA for processes such as transcription, replication, and repair. This is crucial for regulating gene expression and maintaining genomic integrity, and is carried out by a variety of chromatin remodeling complexes that utilize ATP to reposition, eject, or restructure nucleosomes [51]. Several chromatin remodelers have been found to play important roles at regulation defense genes, including the suppression of them under normal developmental stage while activating after pathogen attack [51]. Especially, the interplay between chromatin remodeler and DNAm under biotic stress has been well documented. For instance, the DDM1-mediated gene body DNAm is found to be associated with inducible activation of defense genes [34]. Notably, both DNAm regulators and chromatin remodelers were mainly downregulated at the transition to necrosis stage in our study, suggesting not only the cross-talk but also coordinated downregulation among different epigenetic mechanisms in this susceptible process. Possibly, the silencing of epigenetic regulation, which associated with the downregulation of defense response genes, might be required to facilitate the susceptibility of plants.

Although we found a significant association between DNAm and gene expression alterations in the immune response process of potatoes, other factors, particularly TFs, might also play a pivotal role in regulating transcriptional reprogramming. We identified 952 differentially expressed TFs in this process (Fig. S21a and b, Data S3), including 124 MYB, 91 ERF, 91 bHLH, 61 WRKY, and 46 NAC (Fig. S21c). Some TF families were enriched in these gene clusters with distinct expression patterns (Figs S21d and S22). Notably, the WRKY family, which includes numerous conserved immune regulators such as WRKY11, WRKY22, and WRKY53 [52], was found to be enriched in Cluster-4, which is closely related to the defense response (Fig. S21d). Moreover, the potential targets of these SA and JA/ET signaling TFs, which recognize specific *cis*-elements, (e.g. WRKY to W-box, TGAs to TGA-motif, MYC2 to G-box, and ERF to GCC-box), were found to be enriched in different gene clusters (Fig. S21e–h). Notably, the potential MYC2 targets were enriched in the category of ‘regulation of transcription, DNA-templated’ (Fig. S23), including 9 *ERF*, 3 *WRKY*, and 3 *NAC* genes. This suggests a central role of *MYC2* in regulating the transcriptome reprogramming through its TF targets. This finding is consistent with previous reports and highlights the functional conservation of *MYC2* in plant immune regulation [53, 54].

In summary**,** the transcriptome reprogramming of potato QS9 during the susceptible process to *P. infestans* infection involves both transcriptional and epigenetic regulation. The susceptibility of plants may be characterized by the downregulation of defense response genes, the photosynthesis system, and epigenetic mechanisms, including DNAm regulation. However, the relation between DNAm and gene expression remains controversial. Actually, no definite correlation between them has been reported [10]. We propose that many factors, not limited to the position of DNAm, sequence contexts, ‘reader proteins’ that recognize the DNAm sites [55], other interacting epigenetic mechanisms, and TFs, may collectively influence the effect of DNAm on gene transcription [11]. Notably, DNAm changes are not always related with gene expression regulation. It could play multiple roles in stress response processes. Especially, the silencing of TEs by DNAm might be crucial to maintain genome stability [10, 32, 38, 56]. However, many questions remain to be explored, particularly how these DNAm regulators affect plant immunity in potato, and how different epigenetic mechanisms are coordinated to regulate defense response under pathogen stress. Resolving these questions in the future will be of great significance for elucidating the role of epigenetic mechanisms in plant immunity and facilitating crop resistance breeding.

## Materials and methods

### Plant materials and *P. infestans* isolate

The potato cultivar Qingshu No.9 (QS9), one of the most popular potato varieties in China, was used in this study. The seedlings of QS9 were cultivated at 23°C and under a 16/8 h light/dark cycle for 7 weeks. Subsequently, detached leaves of the QS9 were inoculated with *P. infestans* strain PL6015, which was isolated from a QS9 field in Tianshui, Gansu Province, China. The inoculation assay was performed as previously described [57]. Briefly, the *P. infestans* isolate was cultivated on rye sucrose agar (RSA) medium plates at 16°C–18°C for a period of 9–12 days before zoospore harvesting. The petri dish containing the mycelium was cooled with prechilled distilled water for 2 h in a refrigerator set at 4°C to facilitate zoospore release. To prepare *P. infestans*-infected materials, potato leaves were soaked in zoospore suspension (~5 × 10^4^ spores ml^−1^) for 5 min. Then, the petioles of the detached leaves were wrapped with moistened strips of defatted cotton, and placed in a plastic tray and maintained at 16°C and 100% relative humidity in the darkness to ensure infection. Infected leaves were observed at 0, 6, 12, 24, and 48 hpi. Meanwhile, three biological replicates of the samples were collected, flash frozen in liquid nitrogen, and stored at −80°C.

### RNA extraction and sequencing

Total RNA was extracted from infected QS9 leaves using TRIzol reagent according to the manufacturer’s protocol (Invitrogen, USA). At least 200 mg of plant tissue was used for RNA extraction. RNA libraries were constructed using at least 5 μg of total RNA. Fragmented mRNA was used as a template to synthesize cDNA, which was then sequenced on Illumina HiSeq X Ten machine to generate 150-bp pair-end reads. Library construction and sequencing were performed by SEQHEALTH Corporation in Wuhan, China.

### Transcriptome analysis

Quality control and adaptor trimming were conducted using Trim_galore (v0.4.4). The clean reads were mapped to the potato reference genome (DM6.1, https://spuddb.uga.edu/dm_v6_1_download.shtml) and *P. infestan* reference genome (T30–4, https://ftp.ncbi.nlm.nih.gov/genomes/all/GCF/000/142/945/GCF_000142945_ASM14294v1/) by STAR (v2.7), respectively. Determination of gene expression level and transcript assembly were performed using StringTie (v1.3.6). Gene expression level is measured using FPKM. DEGs were identified by DESeq2 with |log_2_ fold change| ≥ 1 and *P*_adj_ < .05. Coexpression clustering of these DEGs was performed using the R package Mfuzz based on the Euclidean distance and c-means objective functions [21]. GO enrichment analysis of genes in different clusters was performed using Fisher’s exact test, and selected with false discovery rate (FDR) adjust *P*-value <.05.

### The annotation of potato genes

Known defense response related genes, including those in the SA, JA/ET pathways, DNAm regulators, and chromatin remodeling genes, were screened from literature according to the model plants *Arabidopsis thaliana* (https://www.arabidopsis.org/). Orthologous genes in potato were determined by dual-homology searching using Blastp (*E*-value < 1e−5 and with coverage ≥50% of the query sequence), and selected by best match between the two species. Potato *NB-LRR* genes of the three classes TIR-NBS-LRR (TNL), CC-NBS-LRR (CNL), and RPW8-NBS-LRR (RNL) were annotated according to the ANNA database (https://biobigdata.nju.edu.cn/ANNA/) [58]. In ANNA, the potato PGSC DM v4.03 gene models were used, and we identified the best NB-LRR homolog in DM v6.1 by Blastp searching (*E*-value < 1e−5) with the ANNA NB-LRRs as subject. Besides, NB-LRRs were also confirmed by the presence of the NB-ARC domain (PF00931), and the PPR family genes were confirmed by the presence of the PPR domain (PF01535) using Pfam database (*E*-value < 1e−4).

### Transcription factor target analysis

The annotation of potato TF was determined according to the database of PlantTFDB (https://planttfdb.gao-lab.org/). The *cis*-elements of W-box ‘TTTGACY’, TGACG-motif ‘TGACG’, GCC-box ‘AGCCGCC’, and G-box ‘CACGTG’ were used to analyze the potential target genes of WRKY, TGA, ERF, and MYC2 TFs, respectively. The Patmatch scripts (http://arabidopsis.org/cgi-bin/patmatch/nph-patmatch.pl) was used to search these elements in the potential promoter (2 kb upstream of TSS) of potato genes. Fisher’s exact test was used to analyze the enrichment of these elements in each DEG cluster.

### Genomic DNA extraction and whole-genome bisulfite sequencing

Genomic DNA was extracted from at least 200 mg of plant tissues using the DNeasy Plant Mini Kit (Qiagen, the Netherlands) according to the manufacturer’s instructions. A total of 100 ng of genomic DNA, spiked with 0.5 ng of lambda DNA were fragmented by sonication into 200- to 300-bp fragments with Covaris S220 (Thermo Fisher Scientific, Germany). The resulting DNA fragments were treated with bisulfite using the EZ DNA Methylation-Gold™ Kit (Zymo Research, USA) to construct libraries for 150-bp pair-end sequencing on Illumina NovaSeq platform (Illumina, USA) at Novogene Corporation in Beijing, China.

### DNA methylation analysis

The bisulfite nonconversion rate was calculated as the percentage of cytosine sequenced at cytosine reference positions in the unmethylated lambda genome. FastQC (v0.11.5) was used to assess the quality of the raw reads. Trim_galore (v0.4.4) was used to remove adapters and low-quality reads. The DNAm levels of the whole genome and at each cytosine site were analyzed using BatMeth2 pipeline (https://batmeth2-docs.readthedocs.io/en/latest/index.html) with default setting [59]. Generally, only cytosines with coverage exceeding 4× were considered. The global methylation levels of the three cytosine contexts were estimated by the ratio of total C to total (C + T) of the corresponding cytosines that passed the 4× threshold. Differences in DNAm between each postinfection stage and the control (0 hpi) were analyzed using the batDMR module in BatMeth2. Initially, DMCs were selected based on an FDR-adjusted *P*-value < .05, with absolute methylation level differences (|infected stage- 0 hpi|) required to be at least 0.3 for CG, 0.2 for CHG, and 0.1 for CHH contexts. The hypermethylation change is defined as the increase of DNAm from 0 hpi to after-infection stage, while the hypomethylation change is defined as the decrease of DNAm from 0 hpi to after-infection stage. DMRs were identified using a 200-bp sliding window approach. DMRs were selected using the same criteria as for DMCs, with a *P*-value cut-off of <0.01. Additionally, at least five DMCs of the same sequence context and consistent alteration direction were required within a single DMR for CHH types, and at least three DMCs for CG and CHG types. Adjacent DMRs <200 bp apart, with the same sequence context and alteration direction, were merged. The boundaries of each DMR were determined by the two flanking DMCs within that DMR. To analyze the effects of DNAm changes to gene expression, DMGs were identified based on the position of DMRs to the nearby genes [46, 47], including DMRs overlapping with exon, intron, 2 kb upstream of the TSS, and 2 kb downstream of the TES. DMGs were also classified according to the position of DMRs.

### Quantitative real-time PCR analysis

Total RNA was subjected to first-strand cDNA synthesis using the PrimeScript™ RT Reagent Kit with gDNA Eraser (TaKaRa, https://www.takarabio.com). Subsequently, qRT-PCRs were performed with FastStart Universal SYBR Green Master (Roche). The relative expression levels were analyzed using the 2^−△△Ct^ method with the *PiUBC* (Pi08327) and *StEF1α* genes used as internal control for *P. infestans* and potato, respectively. Primers used for qRT-PCR were listed in Table S5.

## Supplementary Material

Web_Material_uhaf297

## Data Availability

The data of this study was submitted to National Genomics Data Center (https://ngdc.cncb.ac.cn/) under the project number PRJCA031393, with the RNA-seq raw reads under the GSA accession CRA019840, and the raw bisulfite-seq reads under CRA019846.
