# Supplementary material for: Genome-wide DNA methylation landscape and its association with the transcriptome reprogramming in potato in response to Phytophthora infestans infection
Source: Hortic Res. 2025 Nov 4;13(2):uhaf297. doi: 10.1093/hr/uhaf297 (PMC12923267; doi:10.1093/hr/uhaf297)
Supplement: Web_Material_uhaf297 [file web_material_uhaf297.zip › Supplymentary file_HR.docx]

## Supplementary Figures and Tables


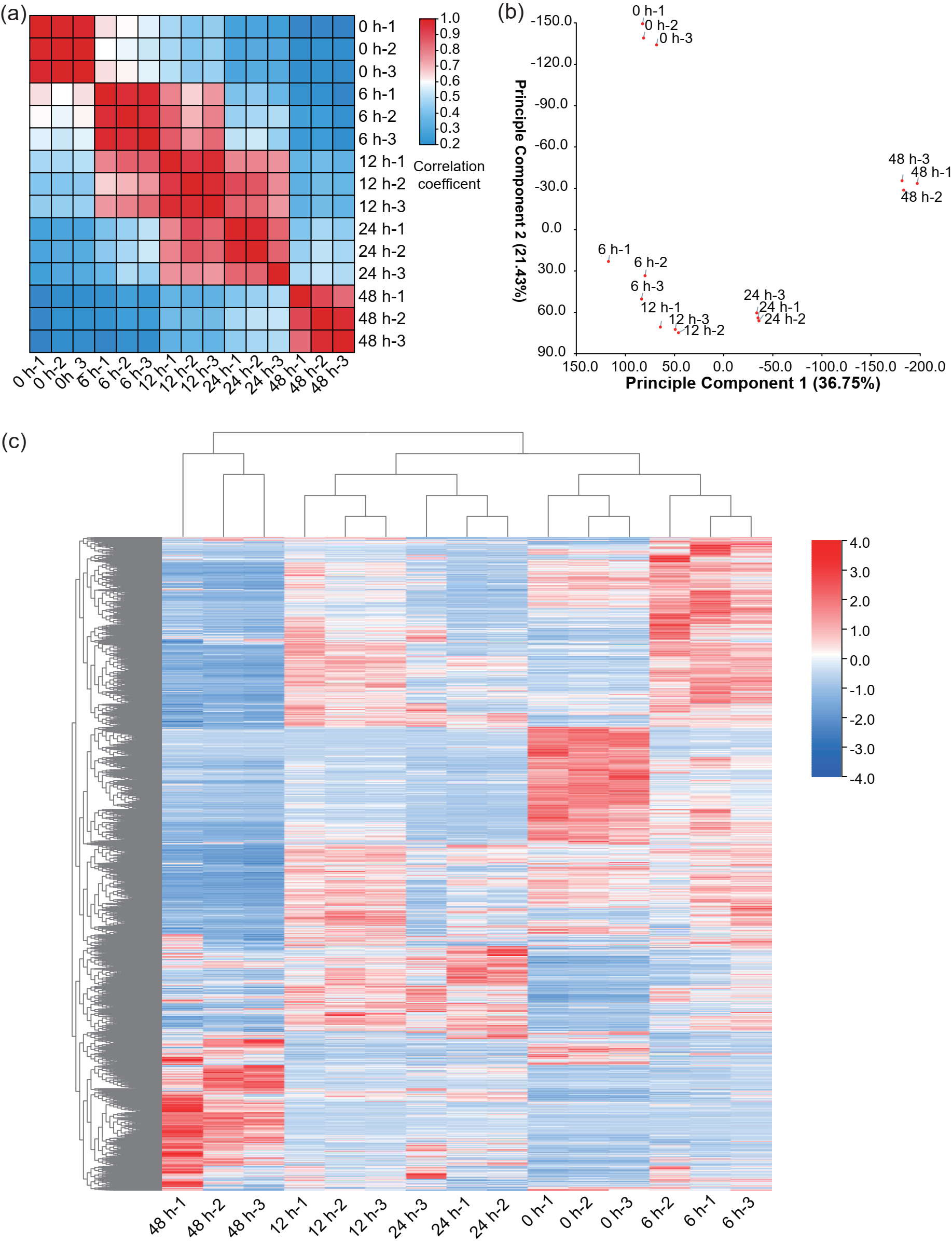


**Figure S1** The high replicability of gene expression between different RNA-seq samples.

(a) Heatmap show the Pearson correlation of gene expression between different samples.

(b) PCA analysis of gene expression level of different samples.

(c) Clustering analysis of gene expression of different samples.


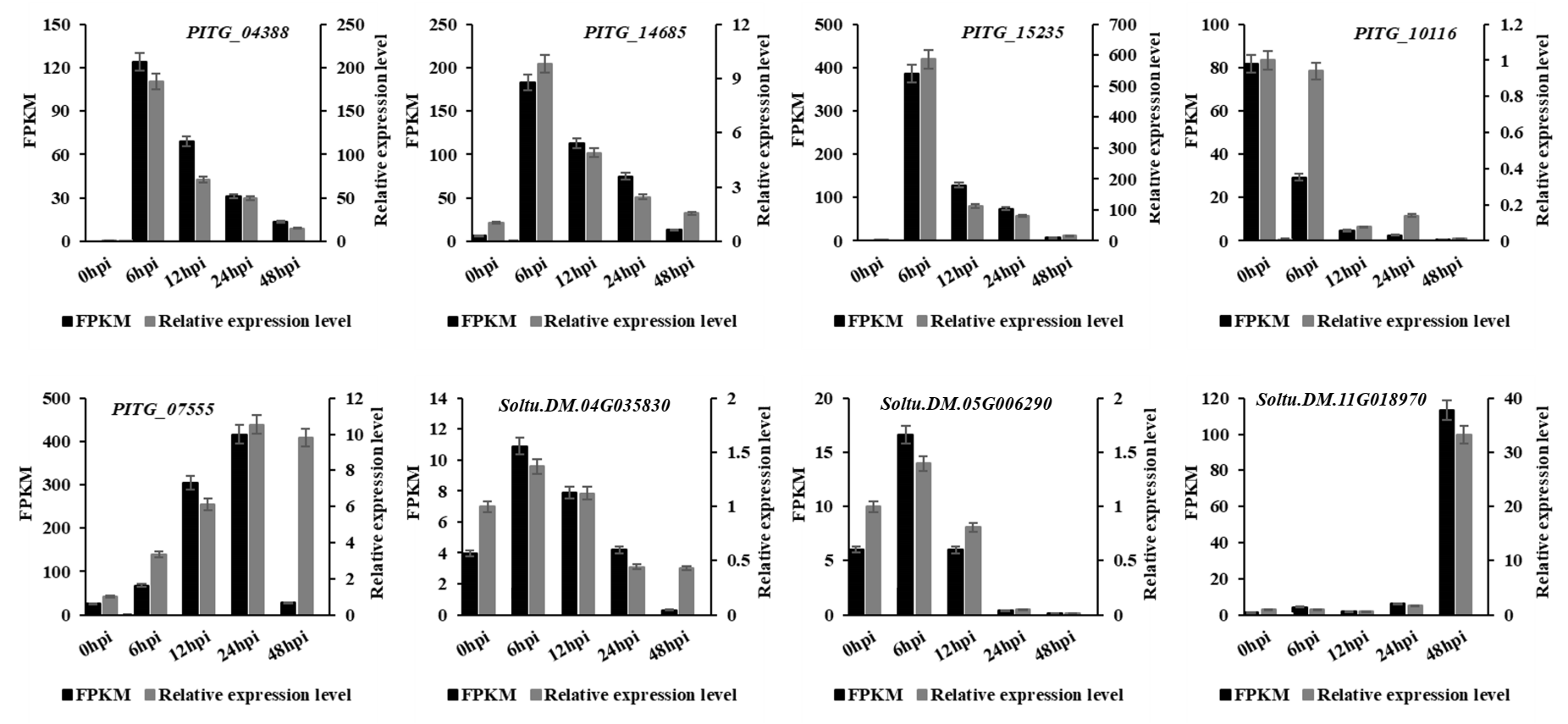


**Figure S2** Verification of gene expression pattern by qRT-PCR.

The ordinate on the left represents the FPKM of the genes, the ordinate on the right represents the relative expression level, and the abscissa represents the infection time.


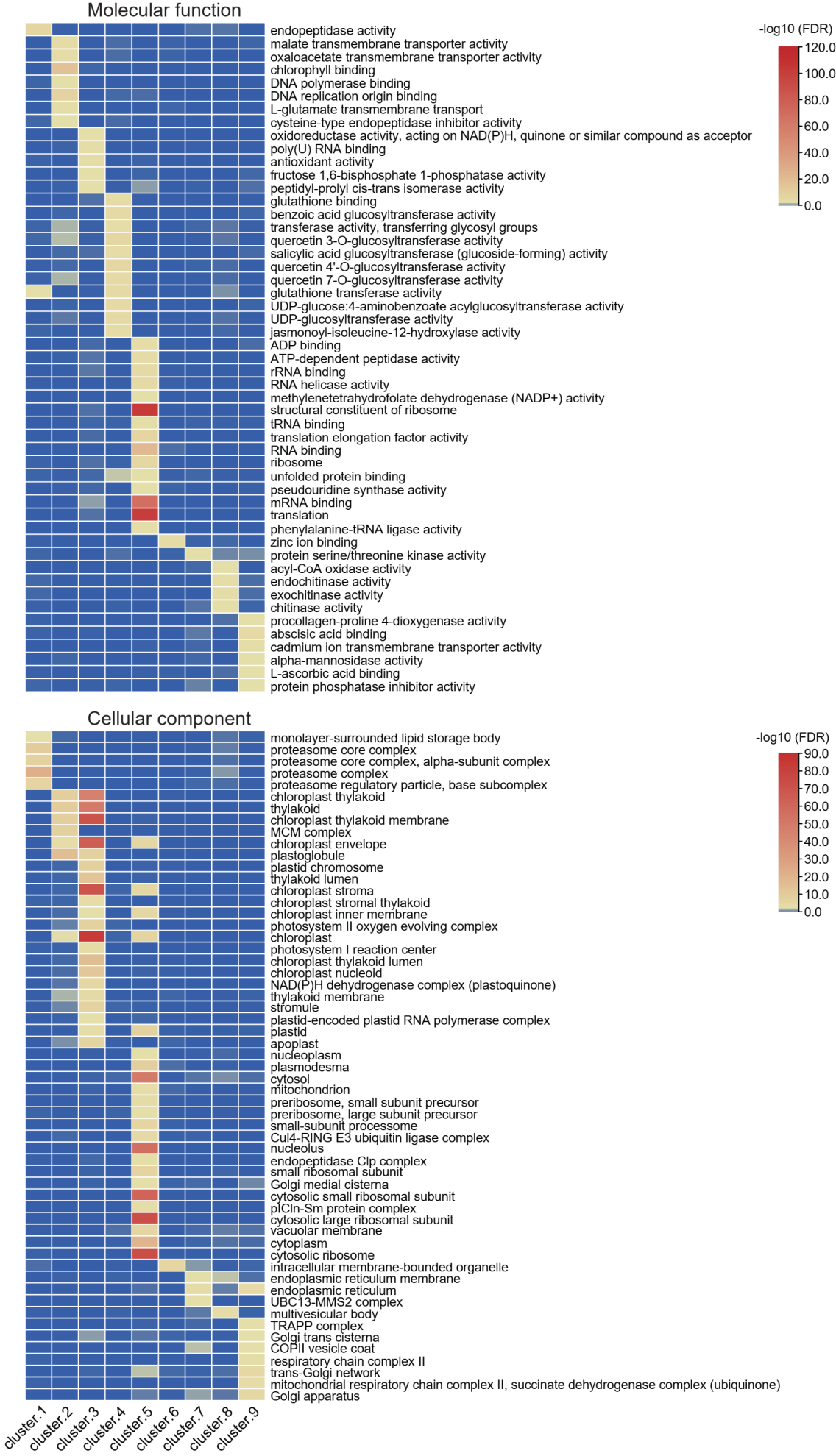


**Figure S3** The enriched molecular function and cellular component of nine clusters with different expression pattern.


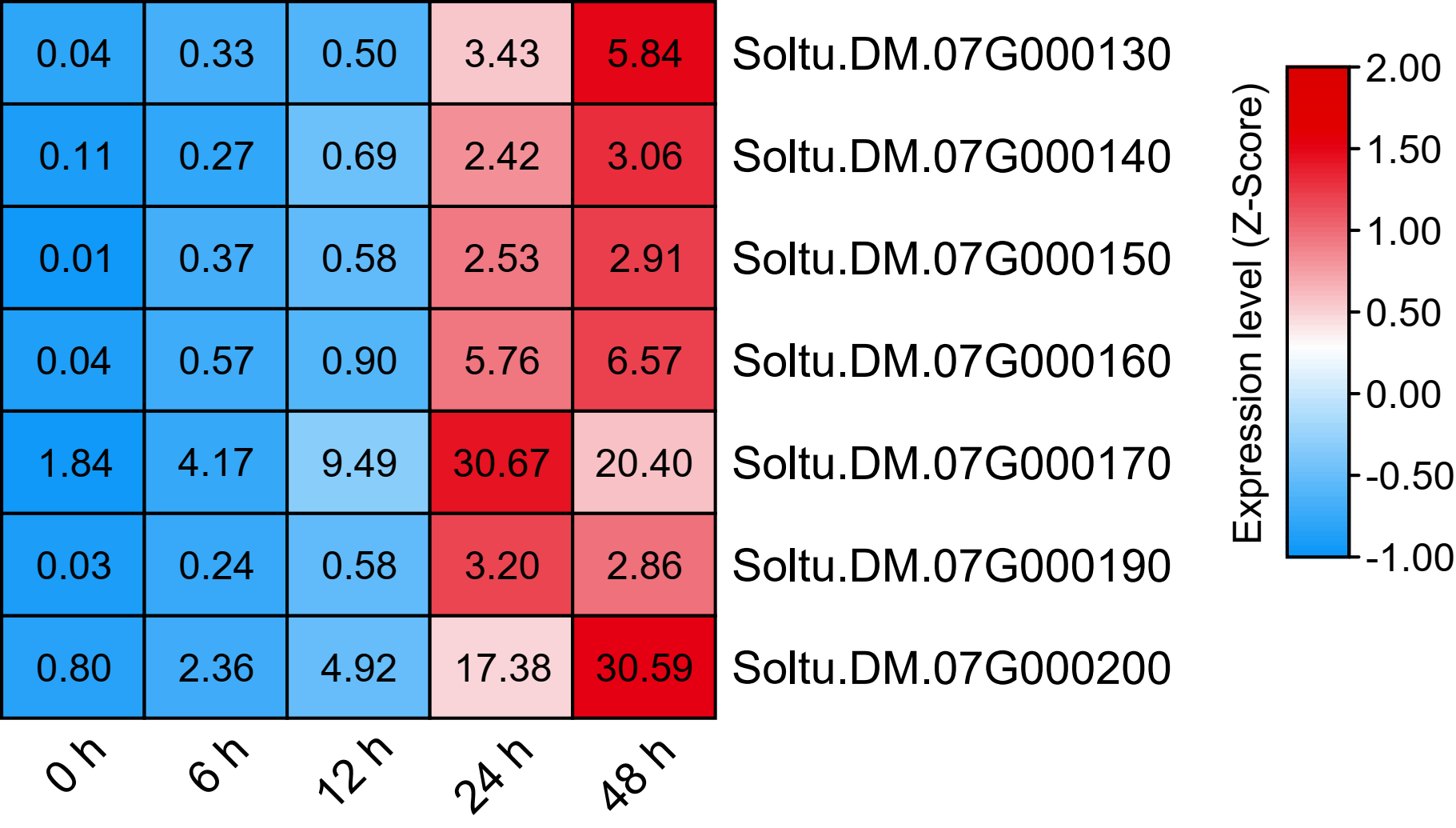


**Figure S4** Expression profile of 7 *ChiC* genes in the infection process.


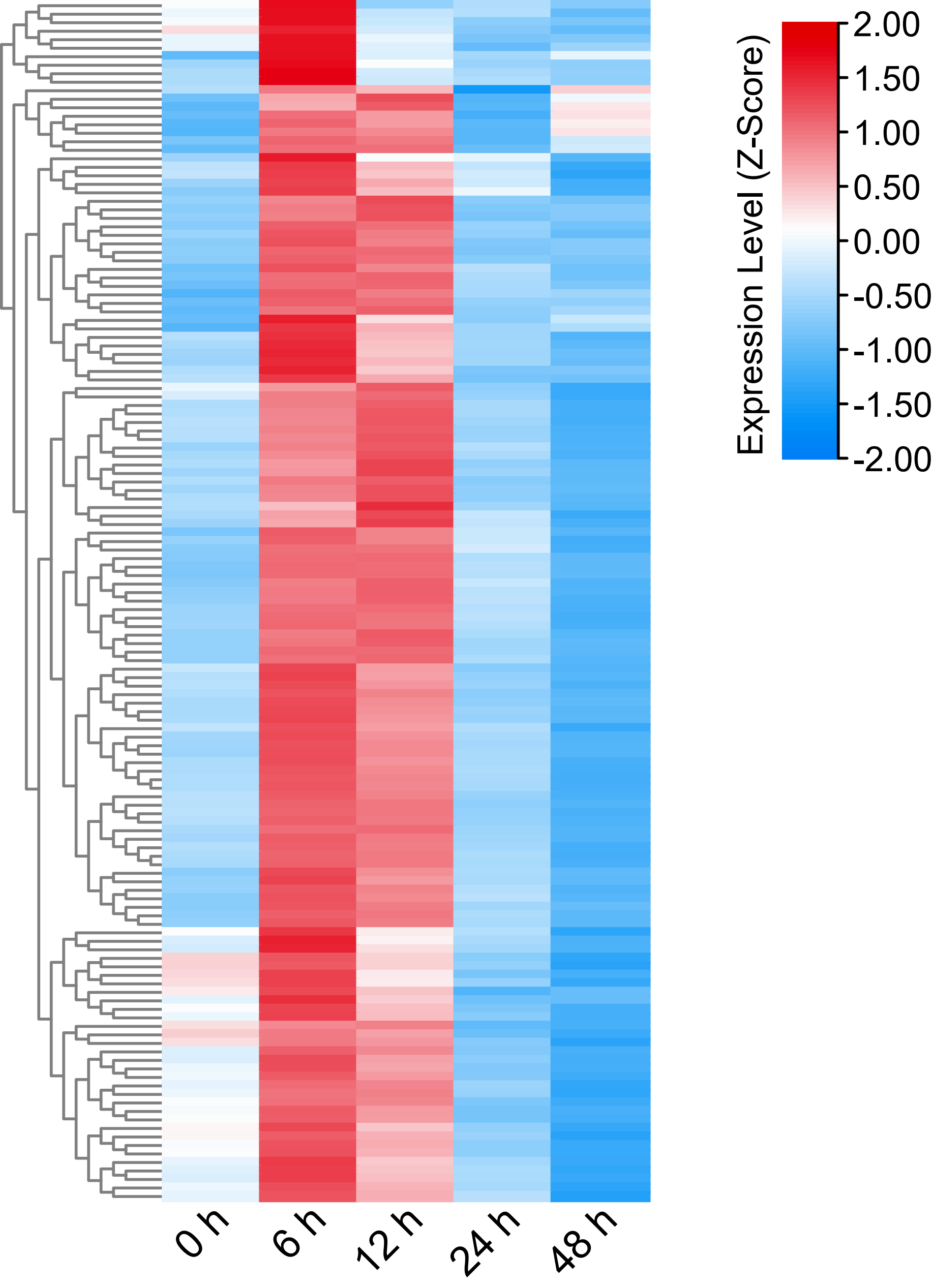


**Figure S5** Expression profile of 141 *PPR* genes in cluster-6.
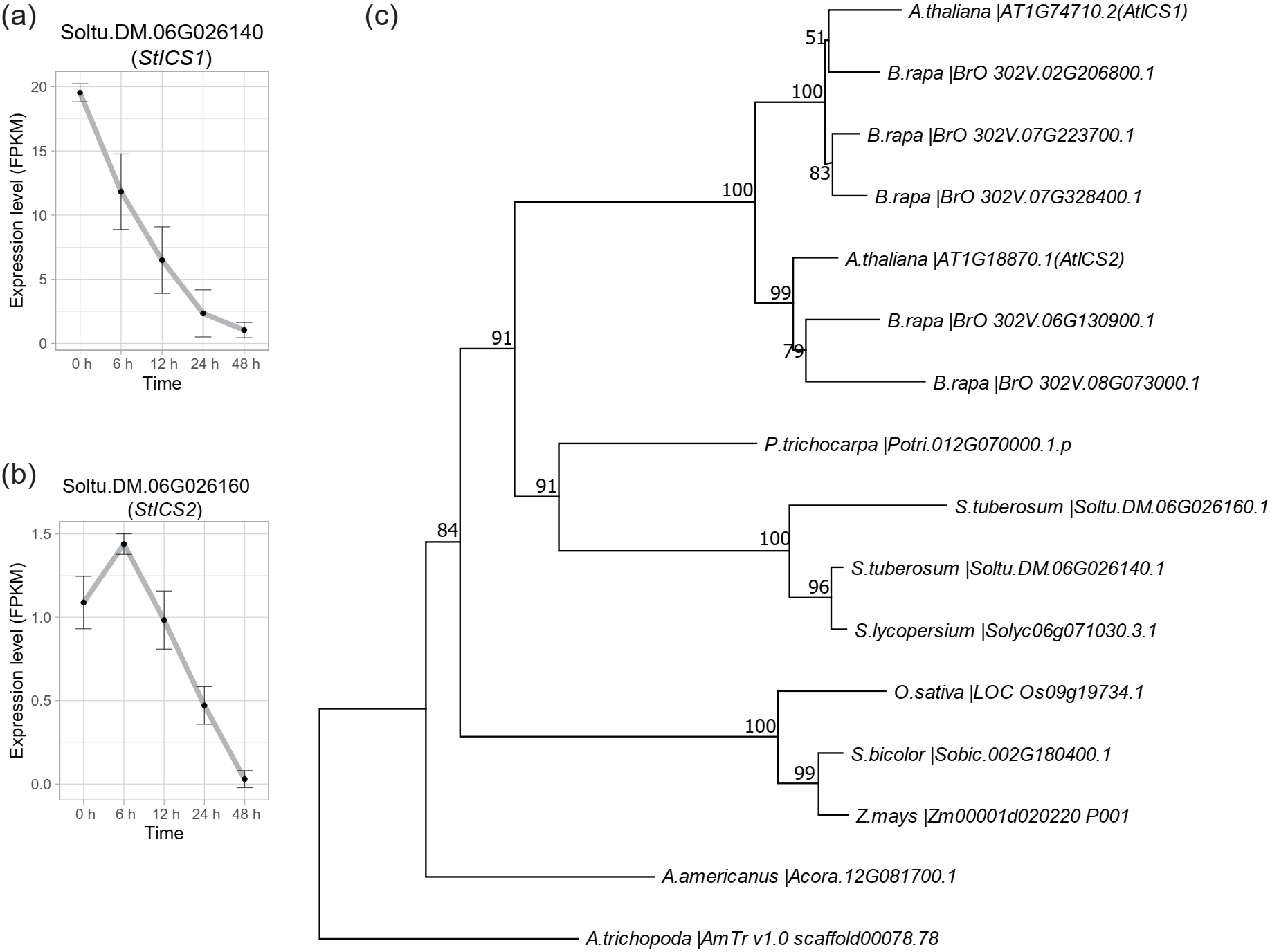


**Figure S6** The SA regulator *ICS* genes in potato

(a-b) The expression pattern of *StICS1* (a) and *StICS3* (b) after the infection of *P. infestans*. (c) Phylogenetic analysis of *ICS* genes in model plants. The phylogenetic tree is constructed based on Neighbor-Joining method by MEGA11 with the number of bootstrap replicates as 1,000. The species and gene id were show in each clade.


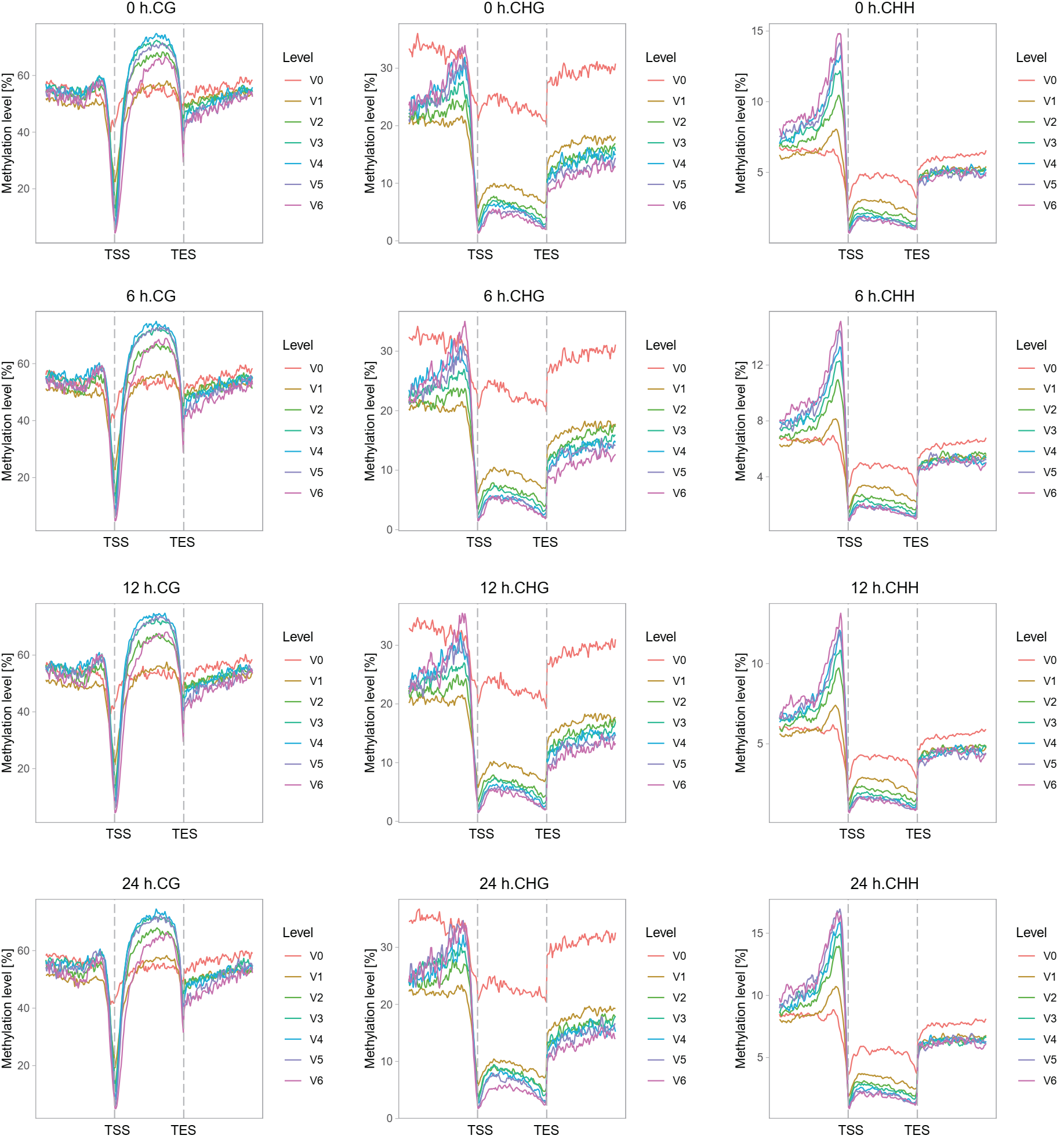


**Figure S7** Comparative analysis between gene expression scales and DNA methylation level for the CG, CHG, and CHH sequence contexts at different stage.

The expression levels were sorted into different scale with V0 as 0, V1 0~1, V2 1~3, V3 3~10; V4: 10~20, V5 20~50; V6: >50. The expression value is normalized as FPKM. The average methylation level of each scale was analyzed with sliding-window approach in the gene body and 2kb flanking regions, with the window and step size as 2.5%, 1.25% of the region length, respectively.


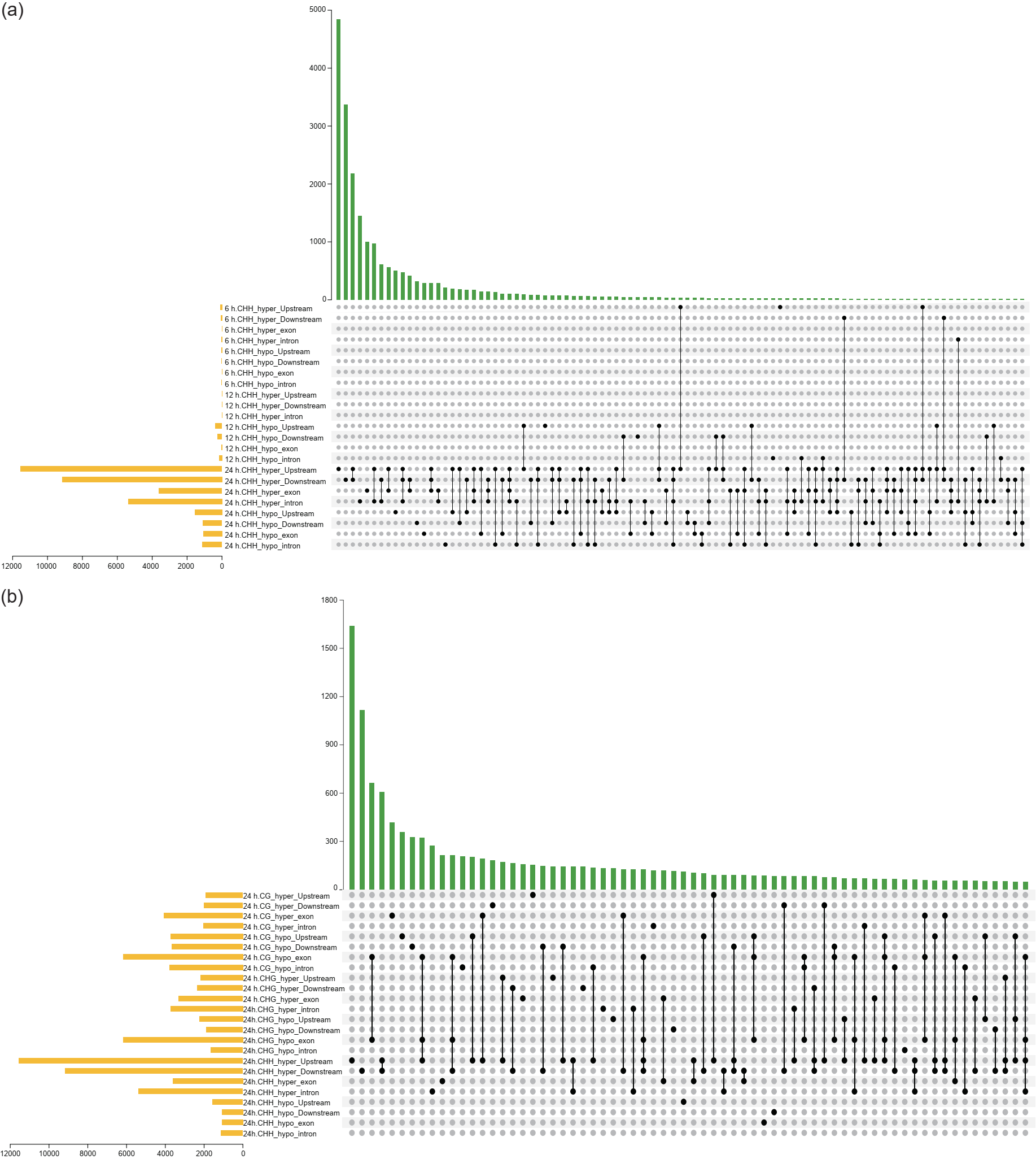


**Figure S8** The co-occurrence of DMRs at gene regions.

(a) The overlapping of CHH-DMR associated genes at different timepoints. (b) The overlapping of DMR-genes of different contexts at 24 hpi.


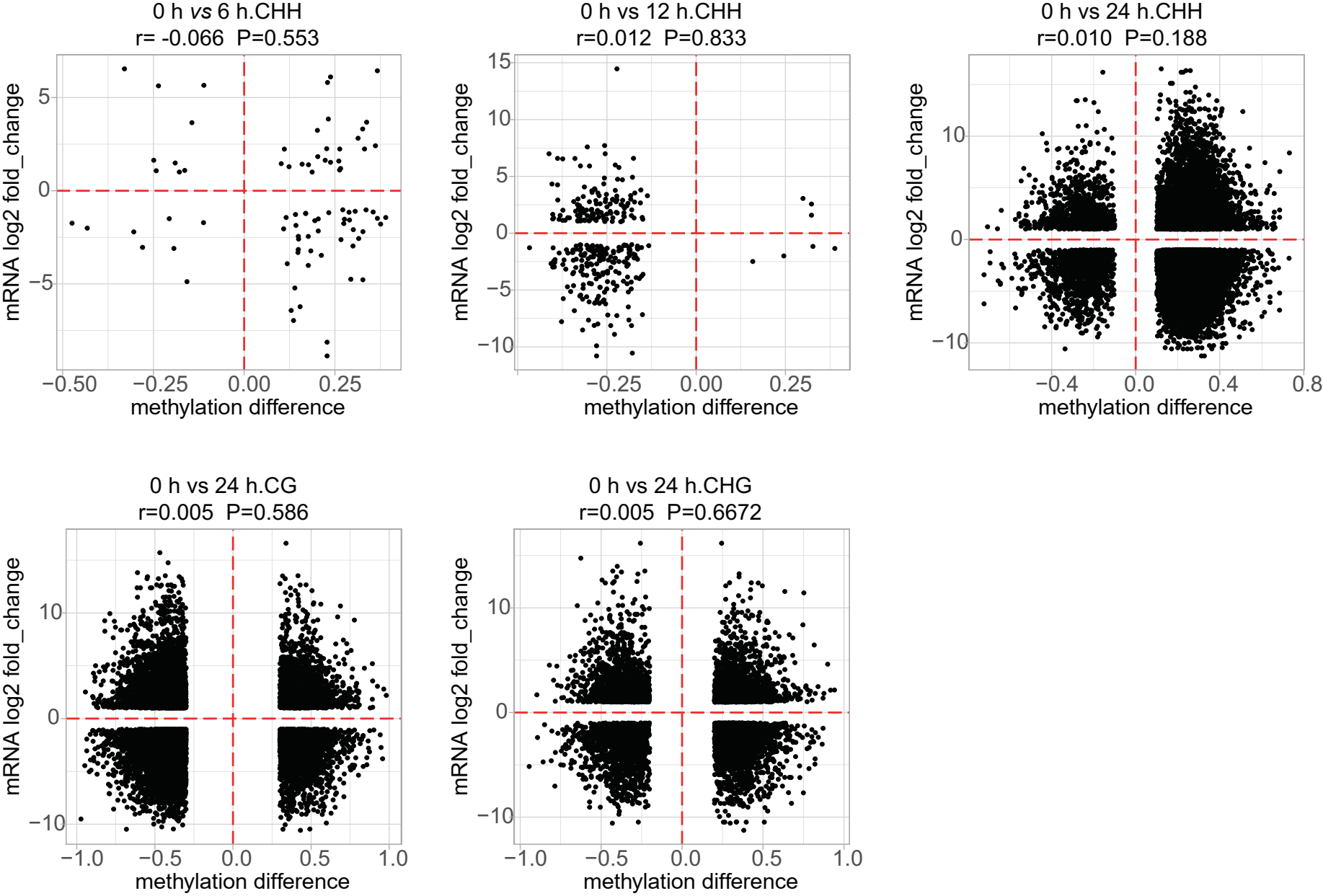


**Figure S9** Correlation between DNA methylation changes in DMRs of different contexts with the expression changes of associated genes.

“r” shows the Pearson’s correlation coefficient. “*P*” shows the significance P-value.


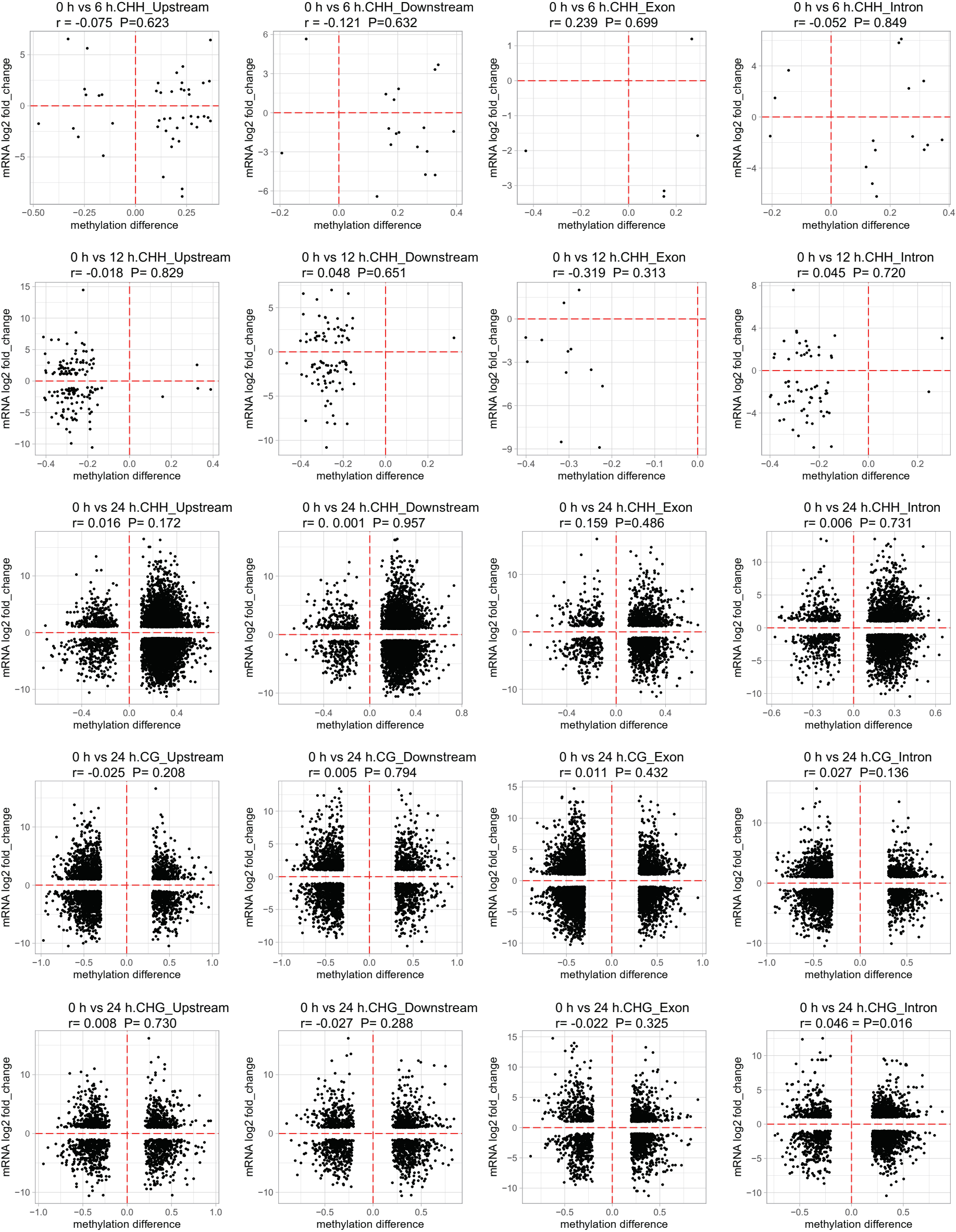


**Figure S10** Correlation between DNA methylation changes in DMRs of different contexts, and different positions with the expression changes of associated genes.

“r” shows the Pearson’s correlation coefficient. “*P*” shows the significance P-value.


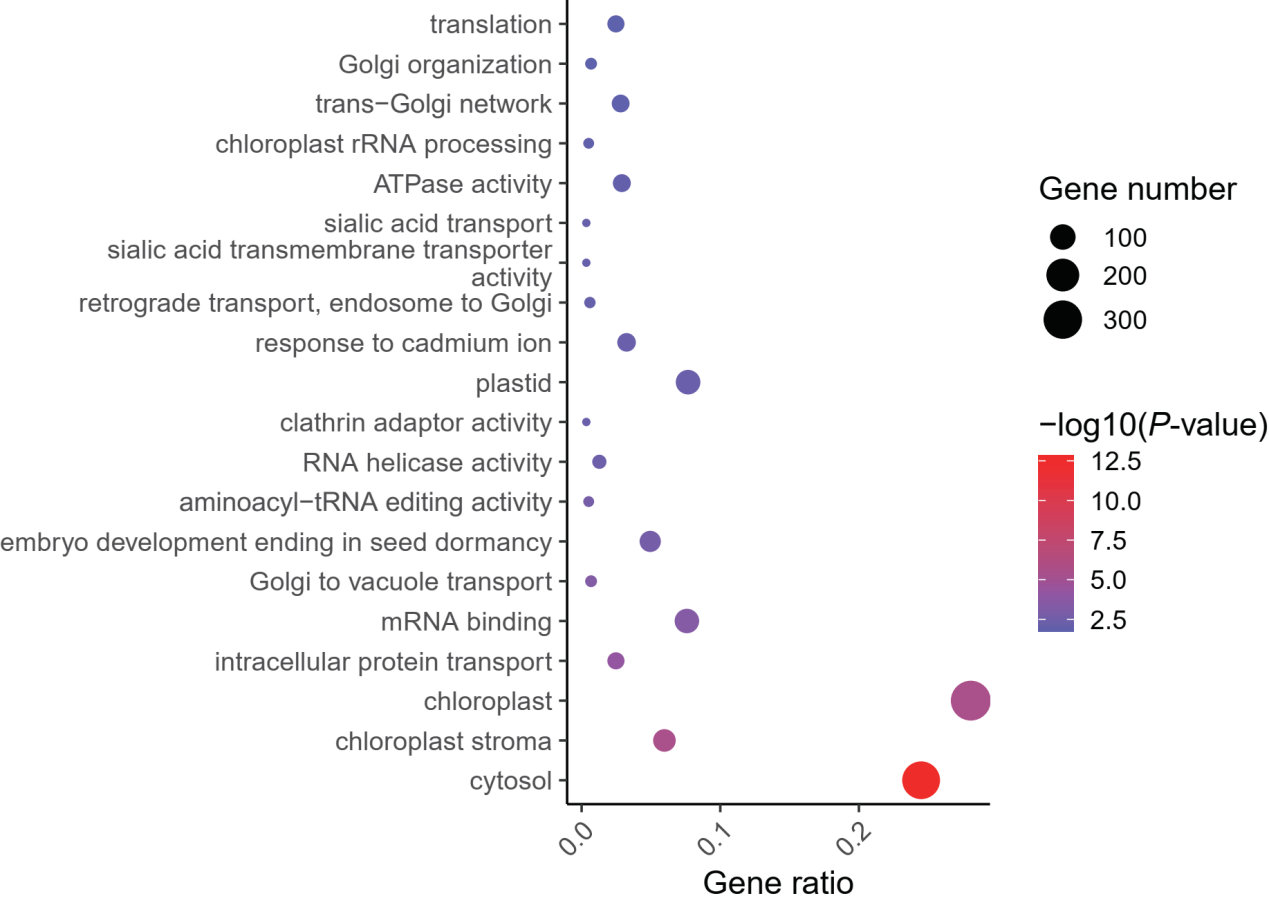


**Figure S11** GO term enrichment of DEGs with hyper-CHG-DMRs at their body.


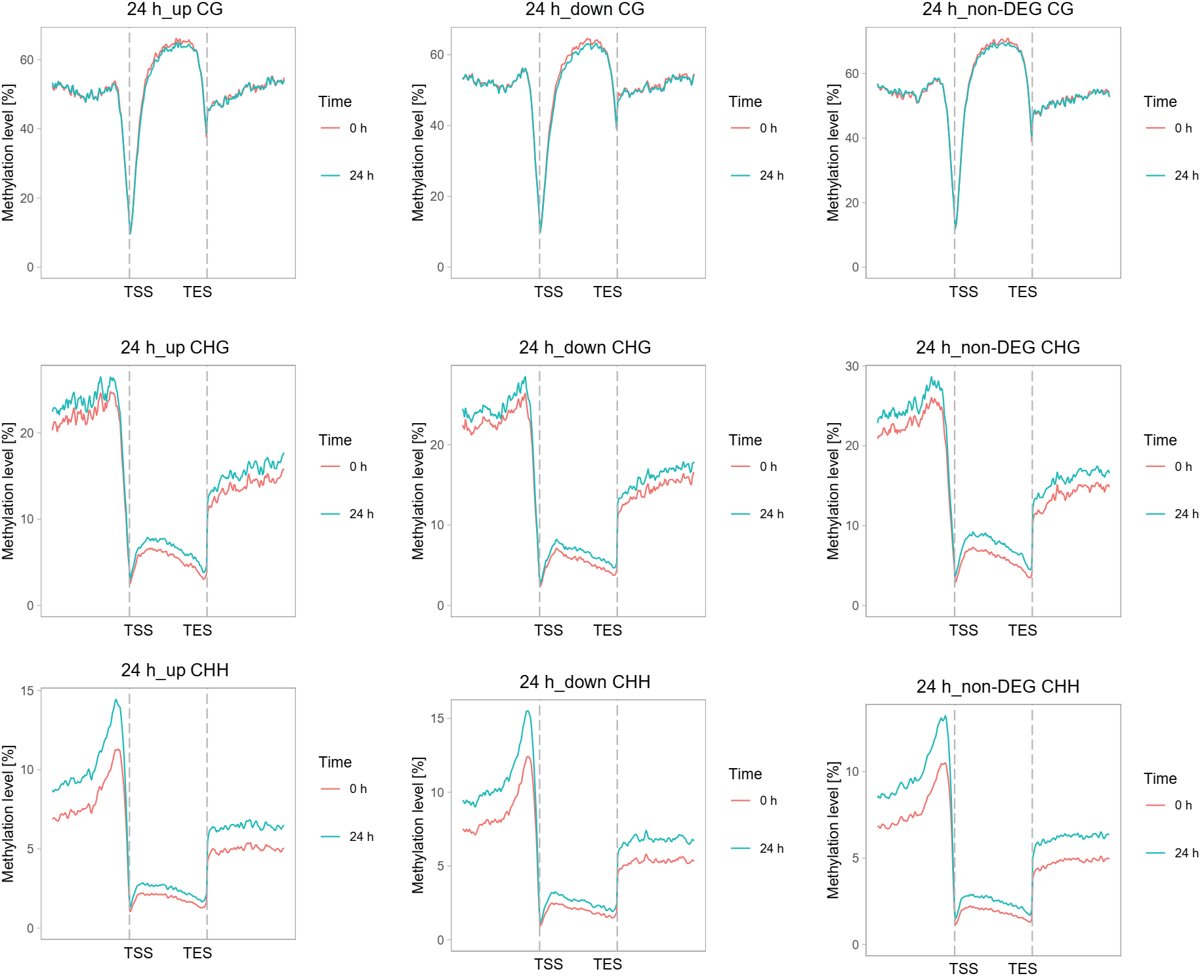


**Figure S12** The pattern of DNA methylation between 24 hpi and 0 hpi at gene regions.

“24 h_up” indicate up-regulate genes at 24 hpi as compared with that at 0 hpi. “24 h_down” indicate down-regulated genes at 24 hpi as compared with that at 0 hpi. “24h_non-DEG” indicate non-differential expressed genes at 24 hpi. The average methylation levels are analyzed by sliding-window approach similar as that in Figure S7.

(a)


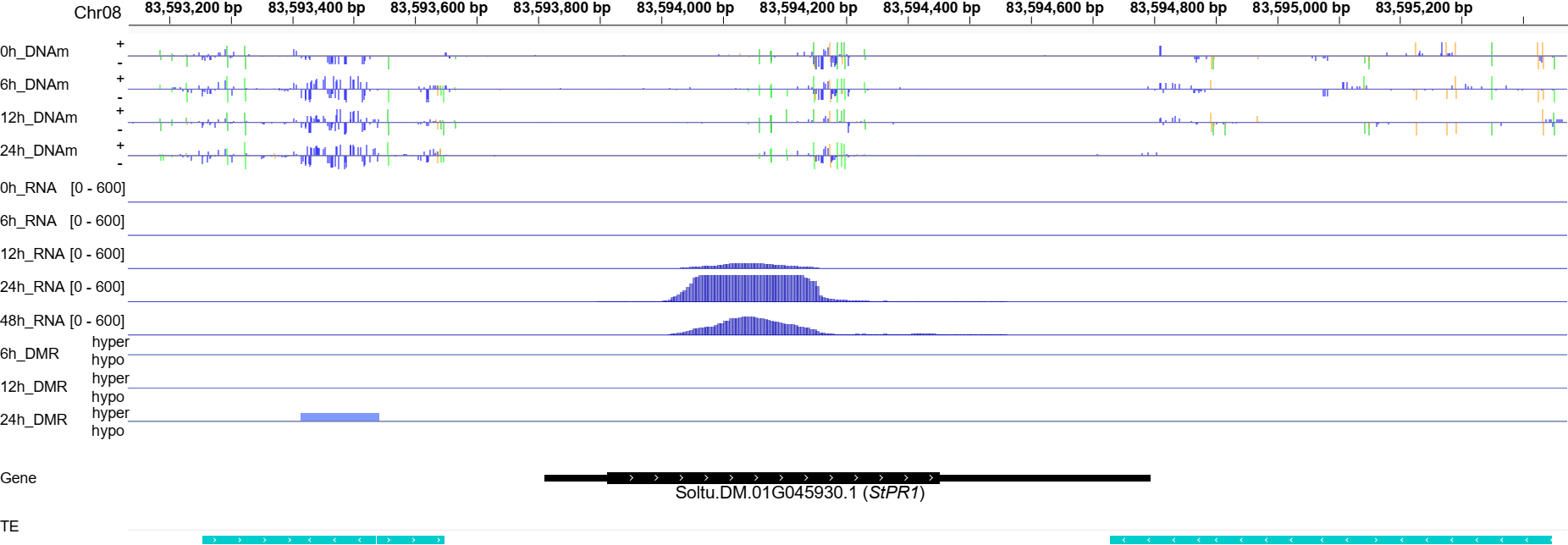


(b)


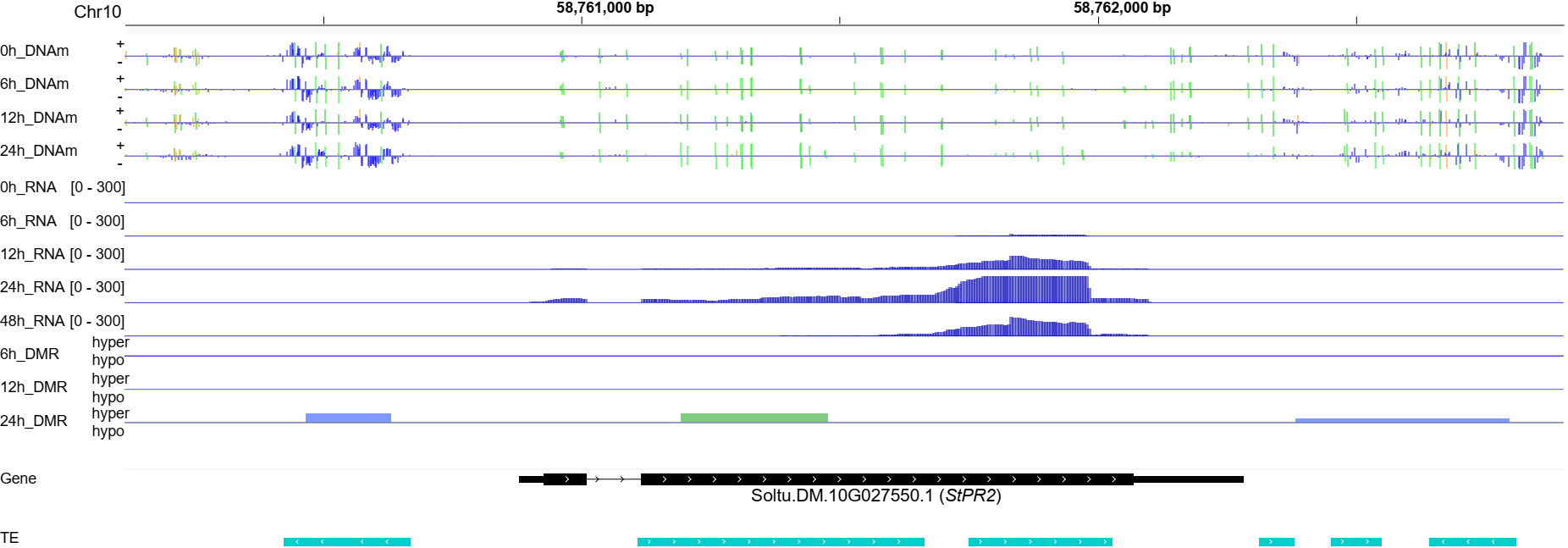


(c)


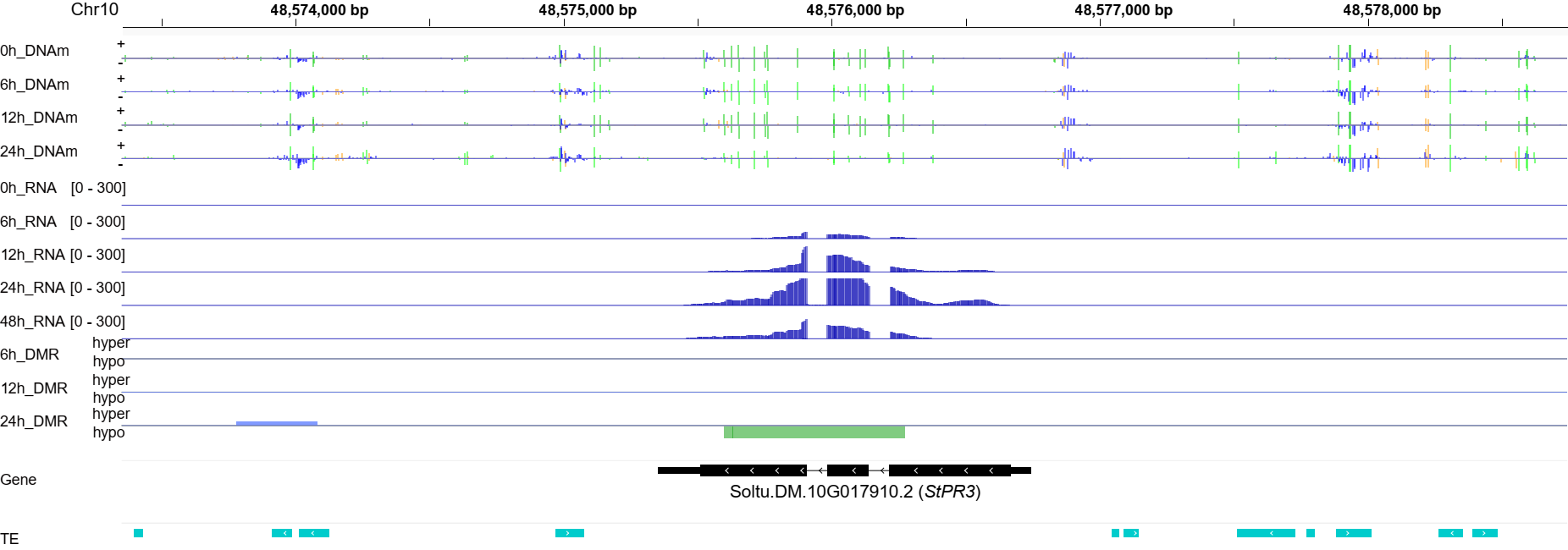


(d)


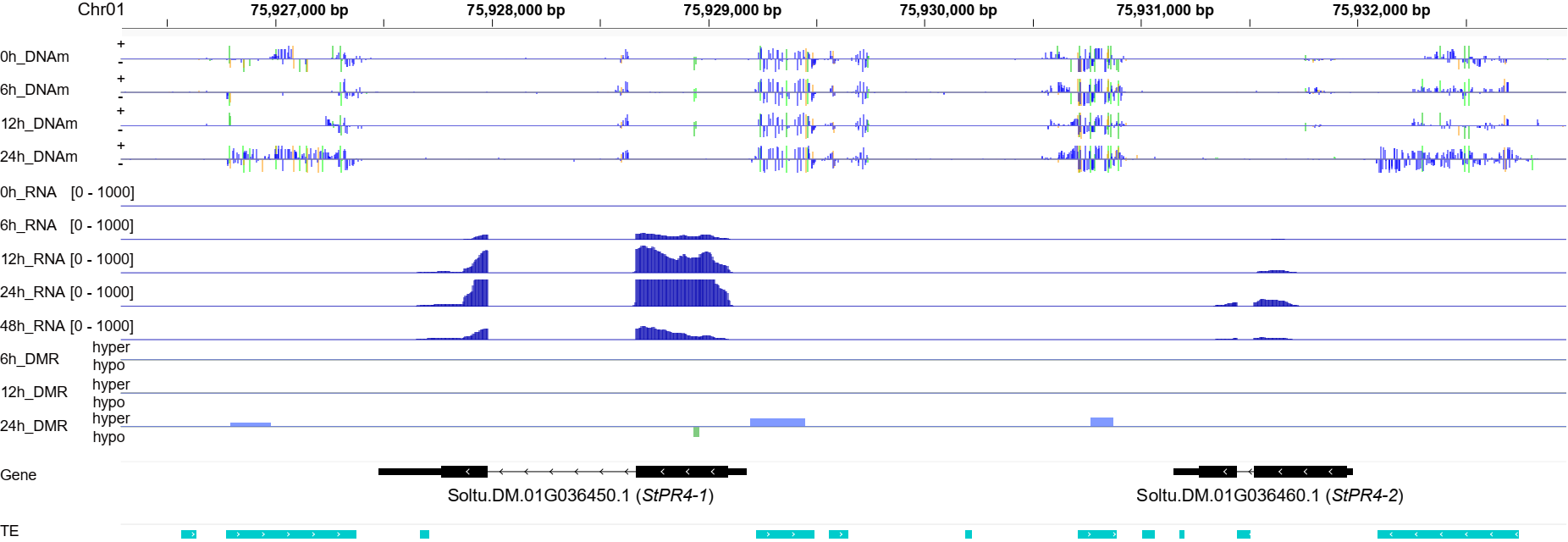


(e)


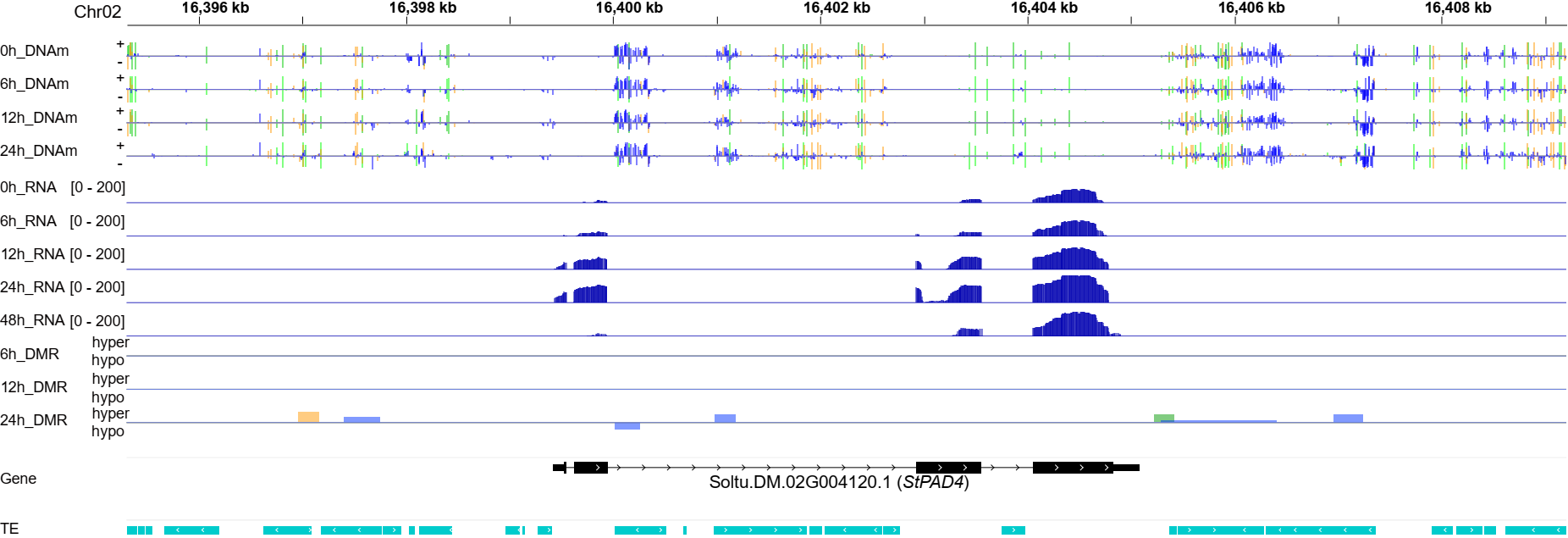


(f)


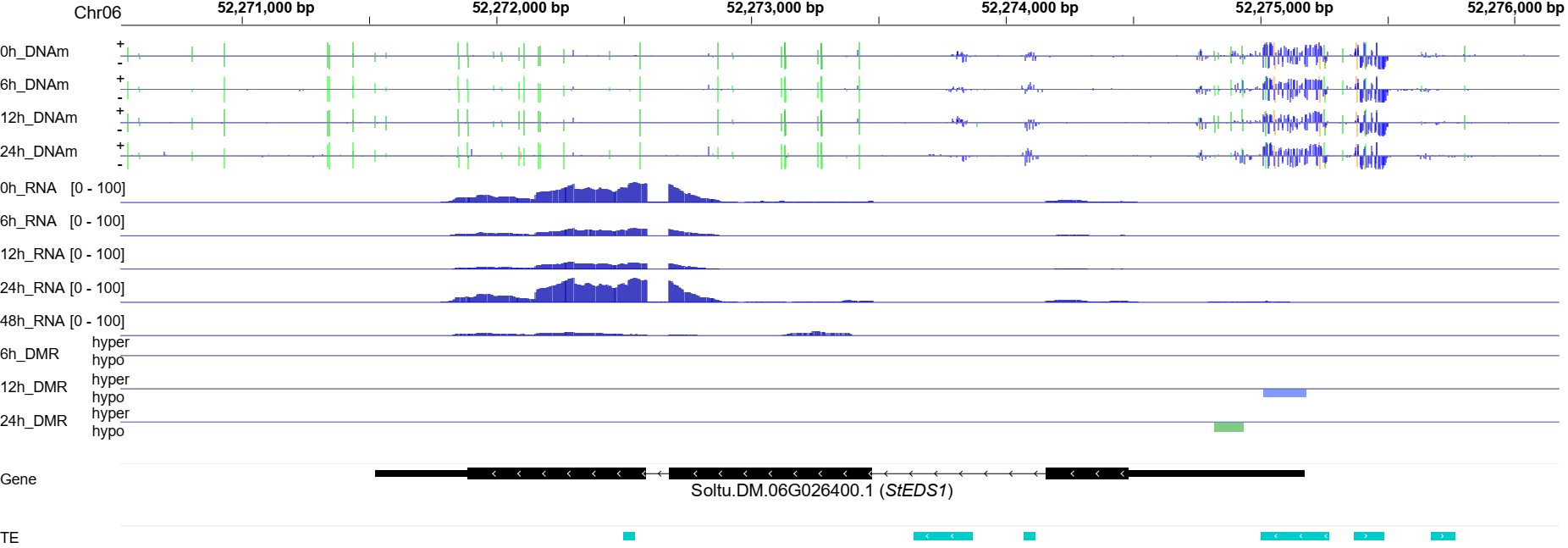


(g)


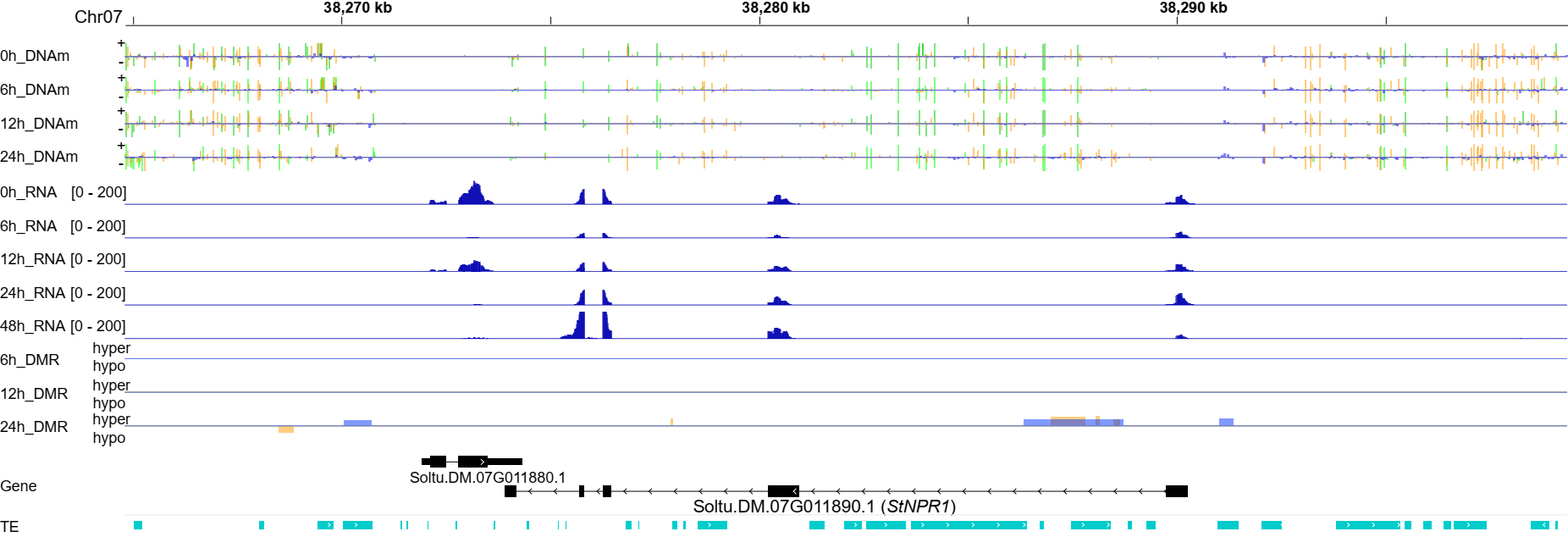


(h)


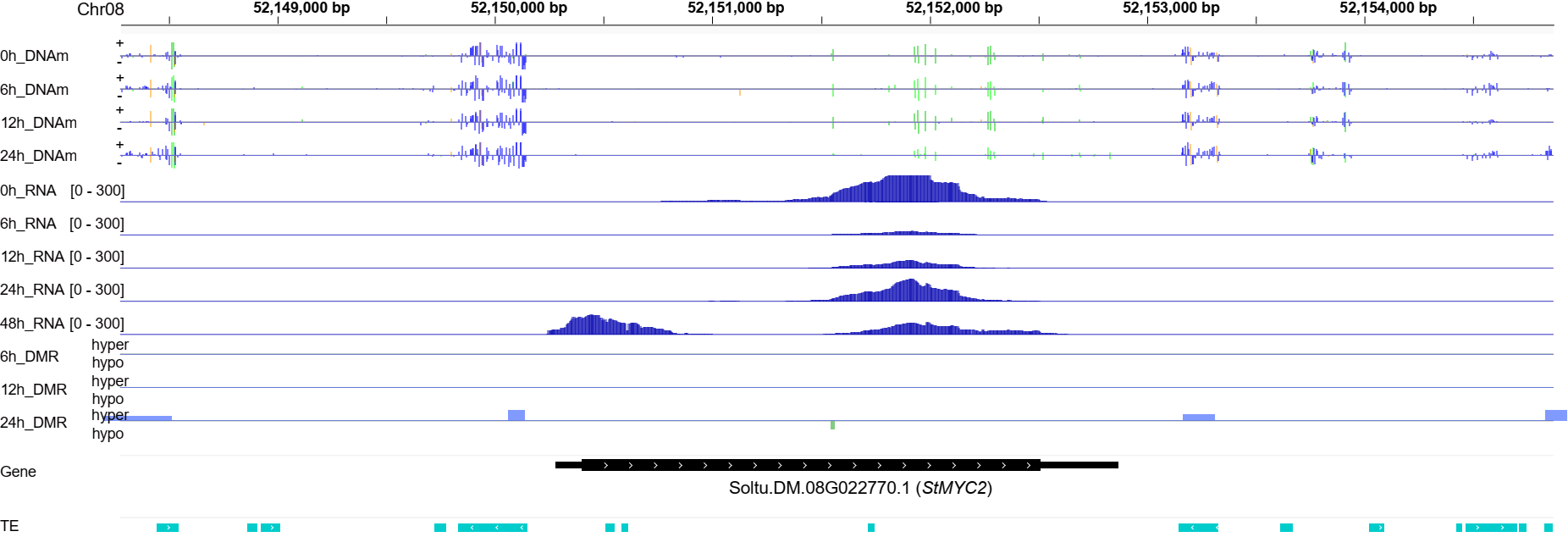


(i)


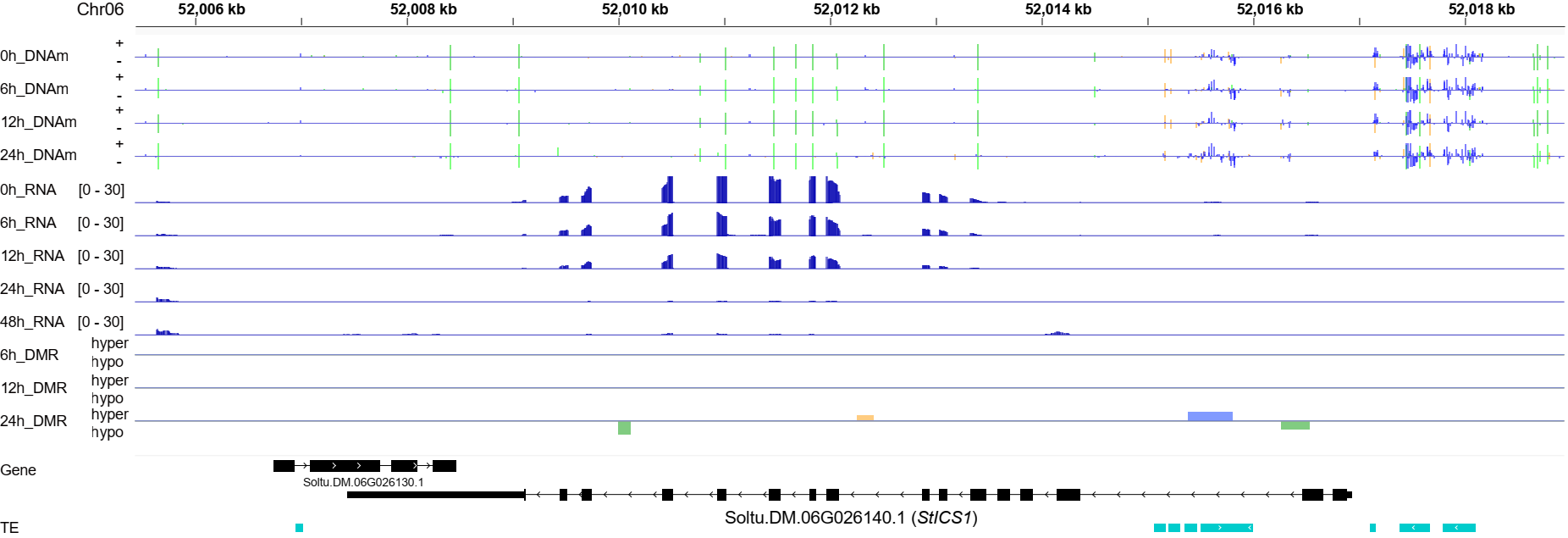


(j)


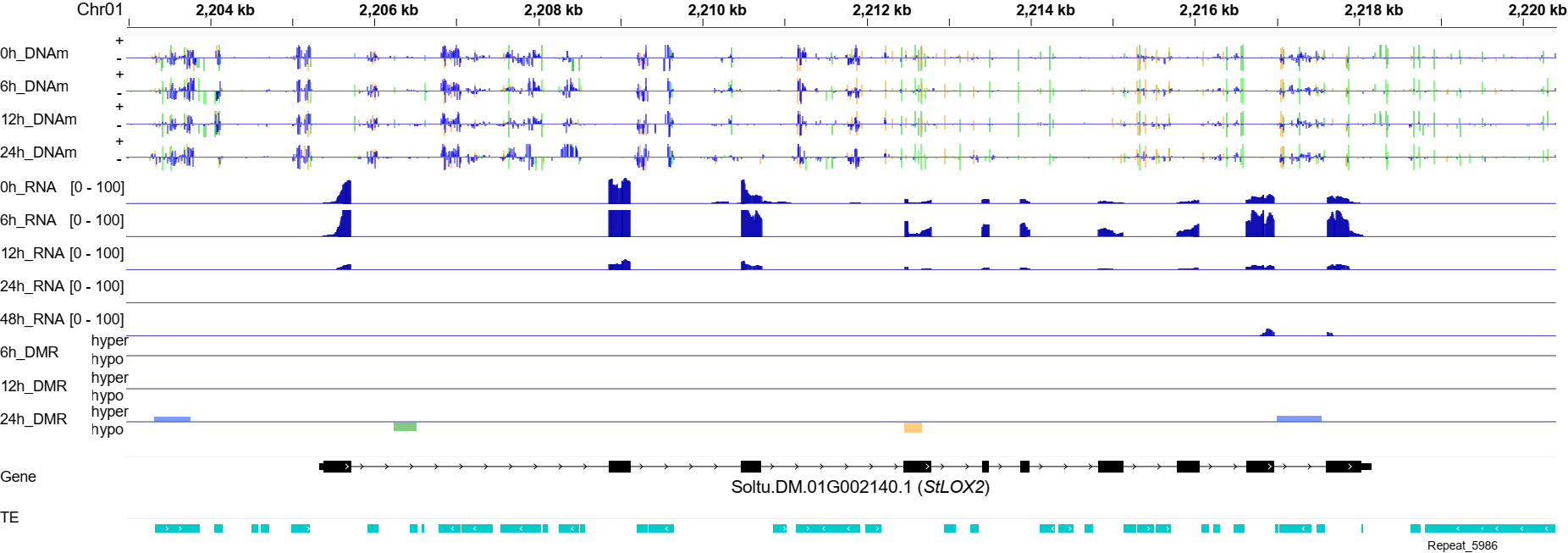


(k)


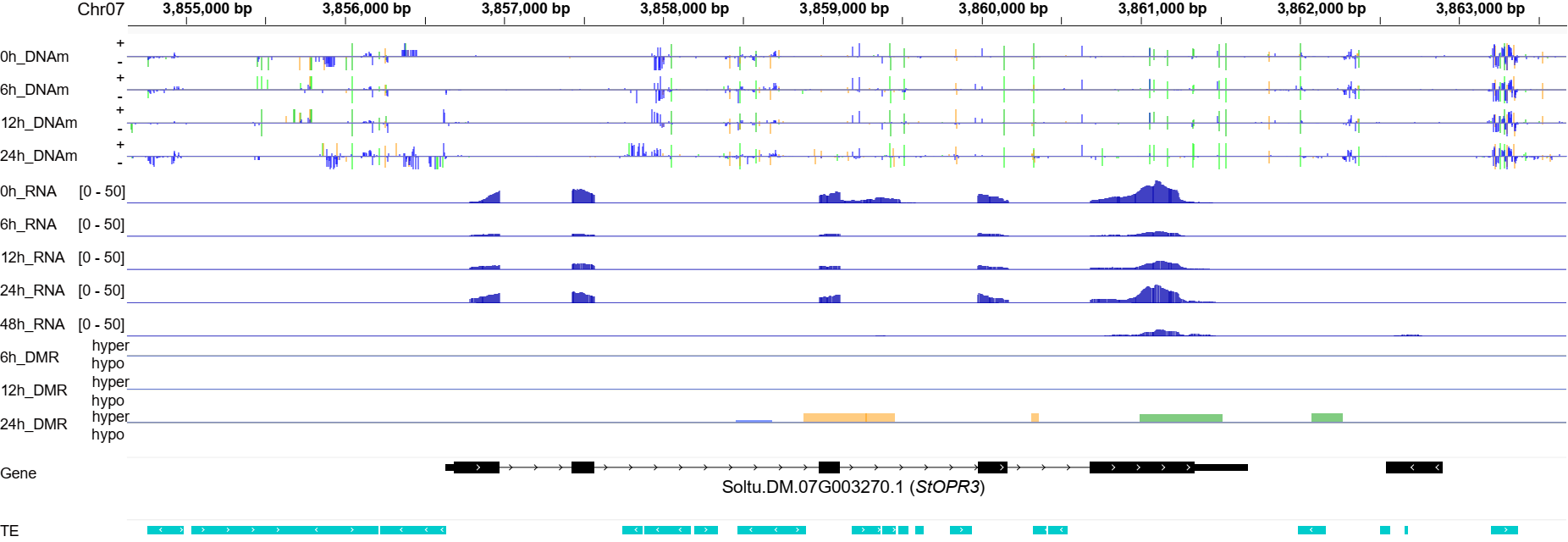


(l)


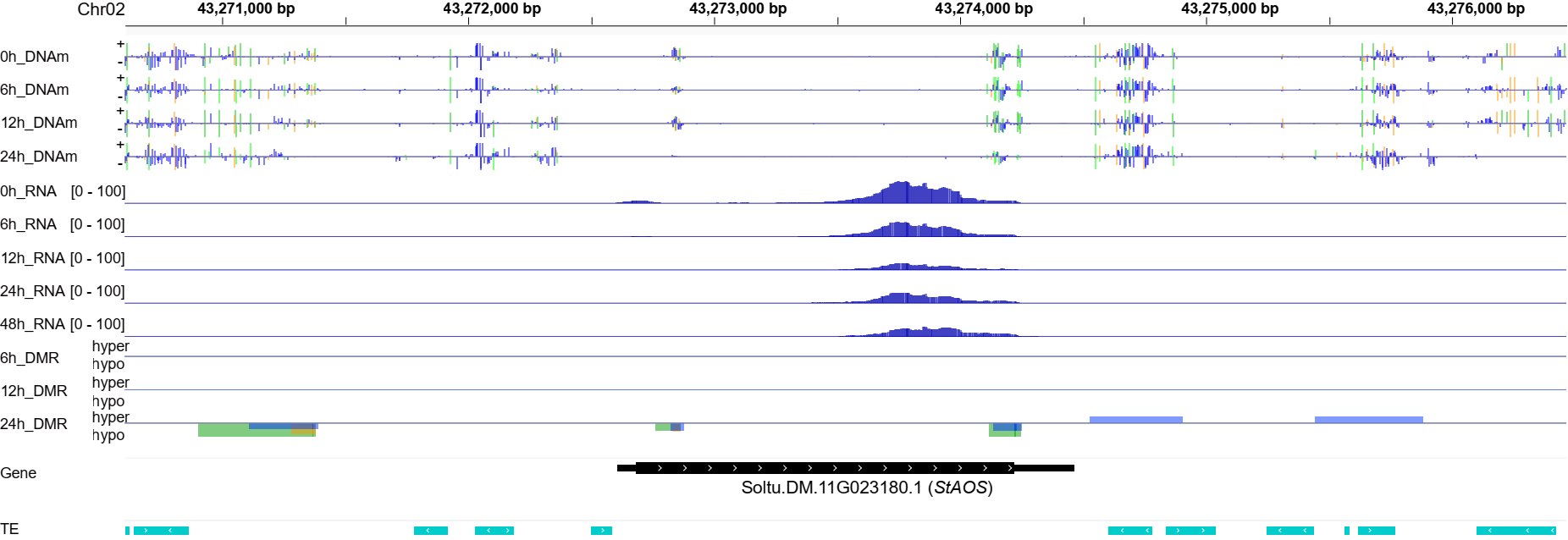


(m)


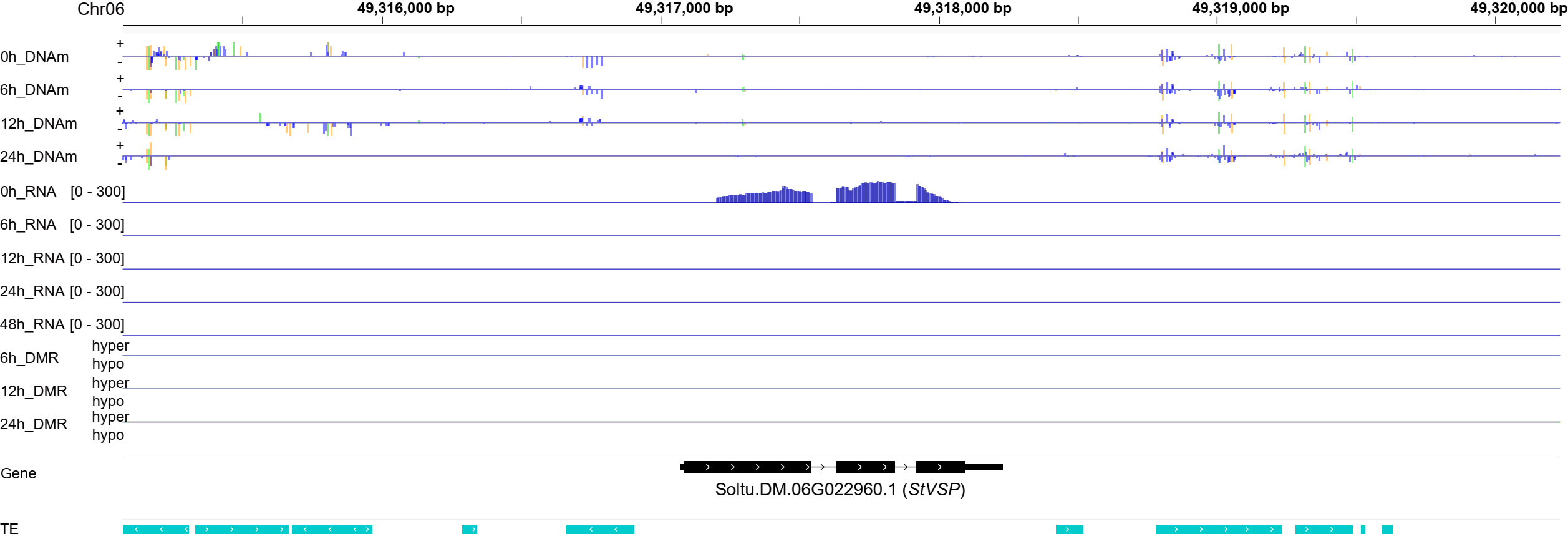


(n)


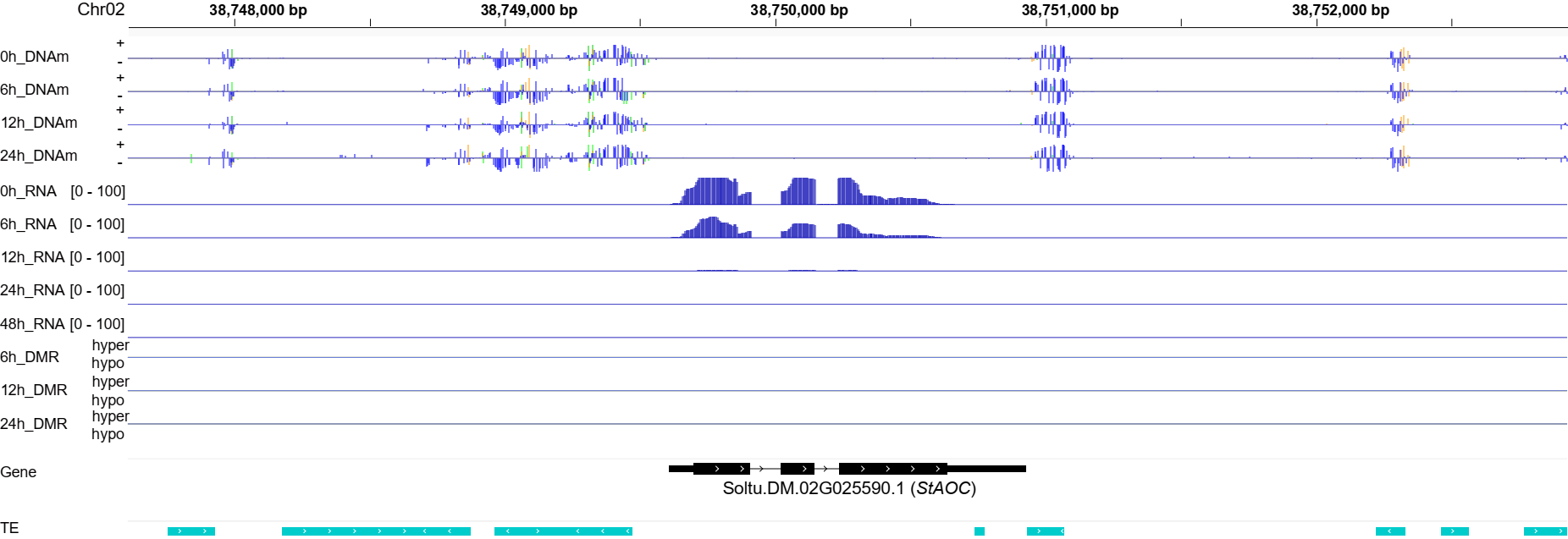


**Figure S13** The expression and DNA methylation pattern around SA, JA/ET signaling related genes.

(a) *StPR1*, (b) *StPR2*, (c) *StPR3*, (d) *StPR4-1* and *StPR4-2*, (e) *StPAD4*, (f) *StEDS1*, (g) *StNPR1*, (h) *StMYC2*, (i) *StICS1*, (j) *StLOX2*, (k) *StOPR3*, (l) *StAOS*, (m) *StVSP*, (n) *StAOC*.

Genome browser screenshot showing the methylation levels, expression levels, and DMRs at different stages. Different cytosine contexts or different DMRs are coded by colors (green for CG, orange for CHG and blue for CHH); The expression level is normalized by reads coverage density as RPM in the square. DMRs are shown as boxes with the width and height to show the region and difference level; the gene structures are showed with black boxes as coding exons, narrow black boxes as untranslated regions (UTRs), lines as introns, and the arrows indicating transcription directions. TEs are presented as cyan boxes with the arrows indicating their strand directions.


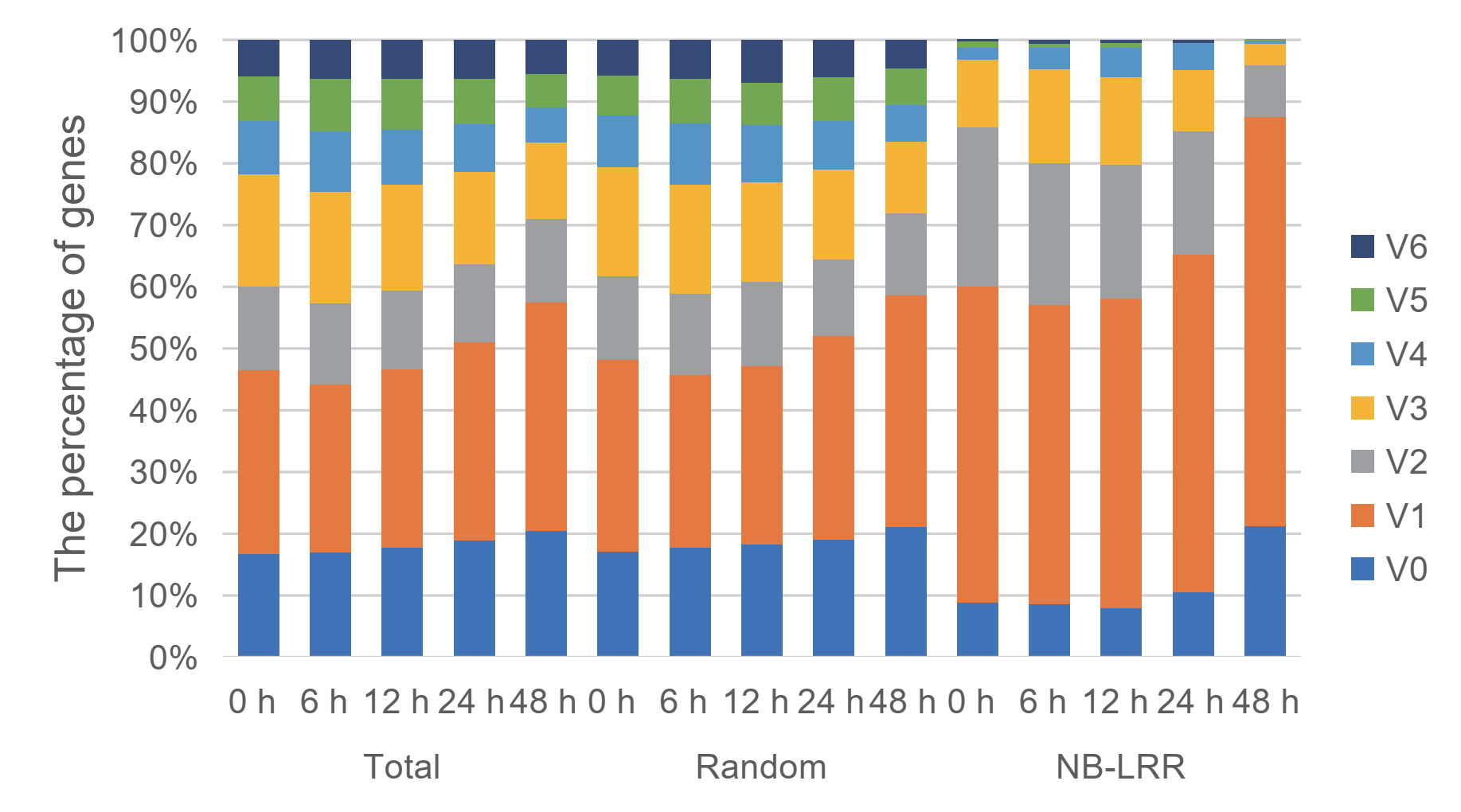


**Figure S14** The expression scale of different types of genes.

“Total” indicates the total 32917 high confident genes of potato reference genome DM6.1. “Random” indicates 2000 randomly selected genes. “*NB-LRR*” indicates the 466 *NB-LRR* genes. The expression levels were sorted as that in Figure S7.


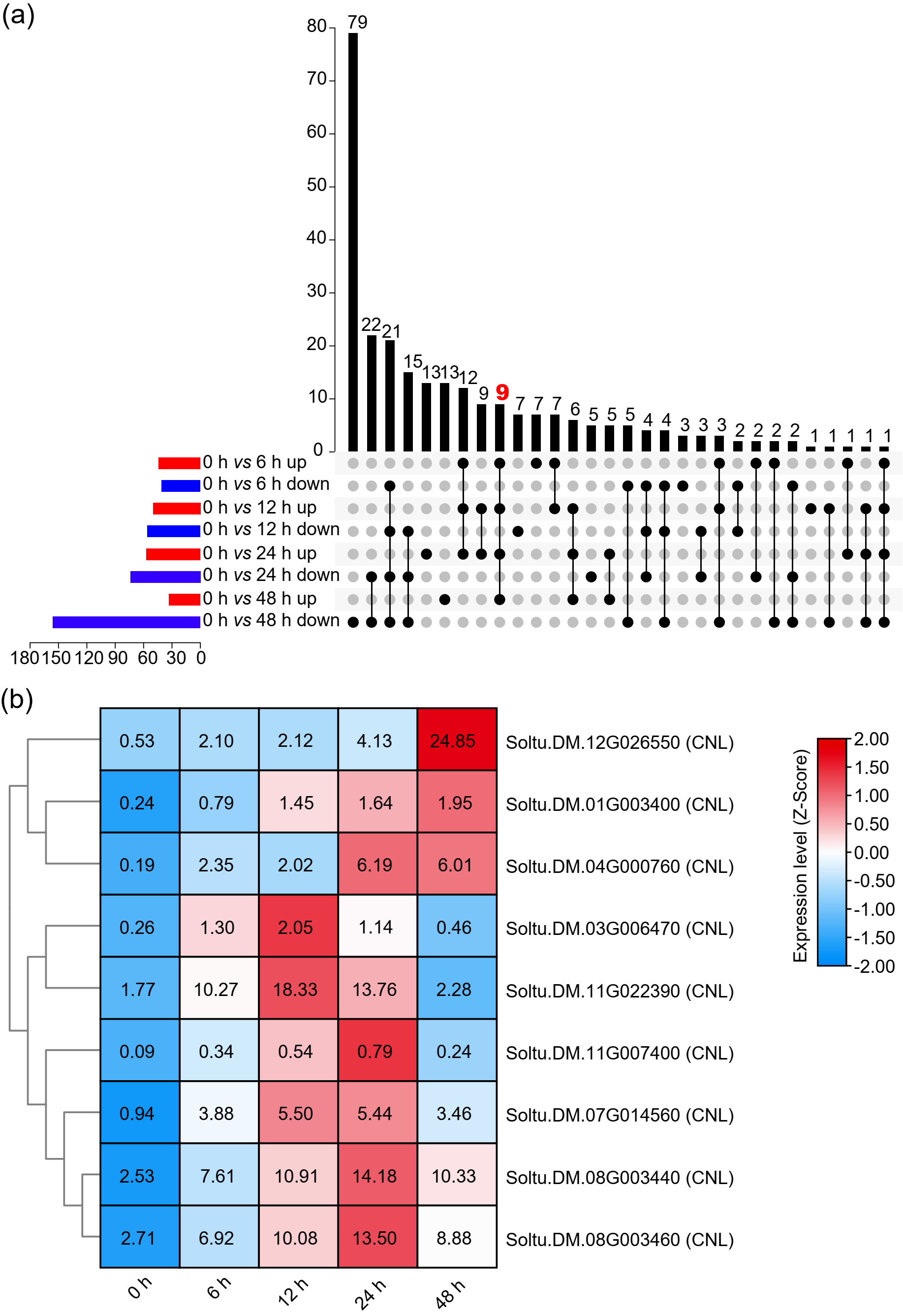


**Figure S15** The expression changes of *NB-LRR* genes in the susceptible process of QS9.

(a) The up-set plot shows the number of differentially expressed *NB-LRRs* in each stage. (b) The 9 *NB-LRRs* with consistent elevated expression as compared to the non-infected stage.


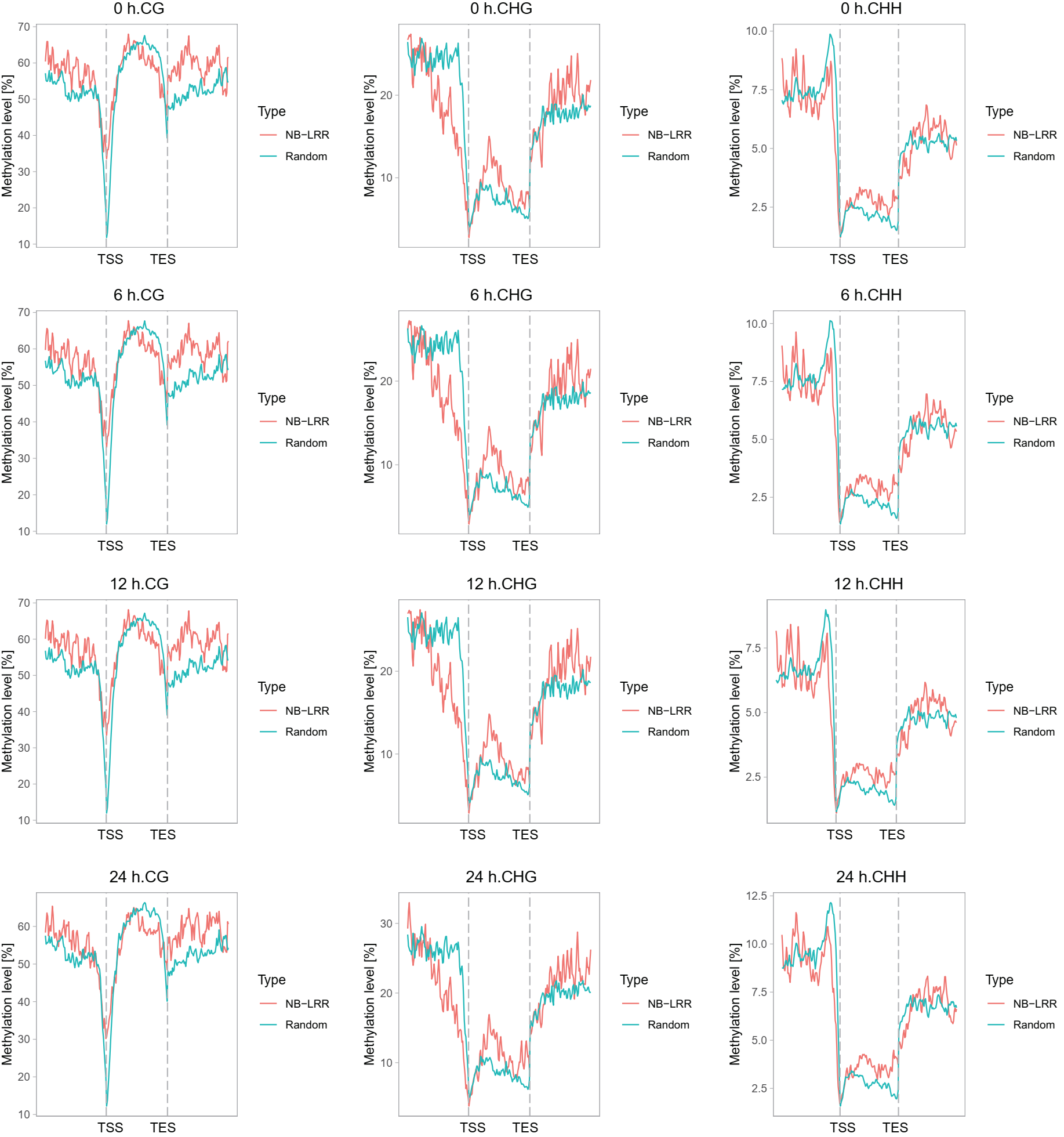


**Figure S16** Comparison of DNAm pattern between *NB-LRR* and randomly selected genes.

“Random” indicates 2000 randomly selected genes.


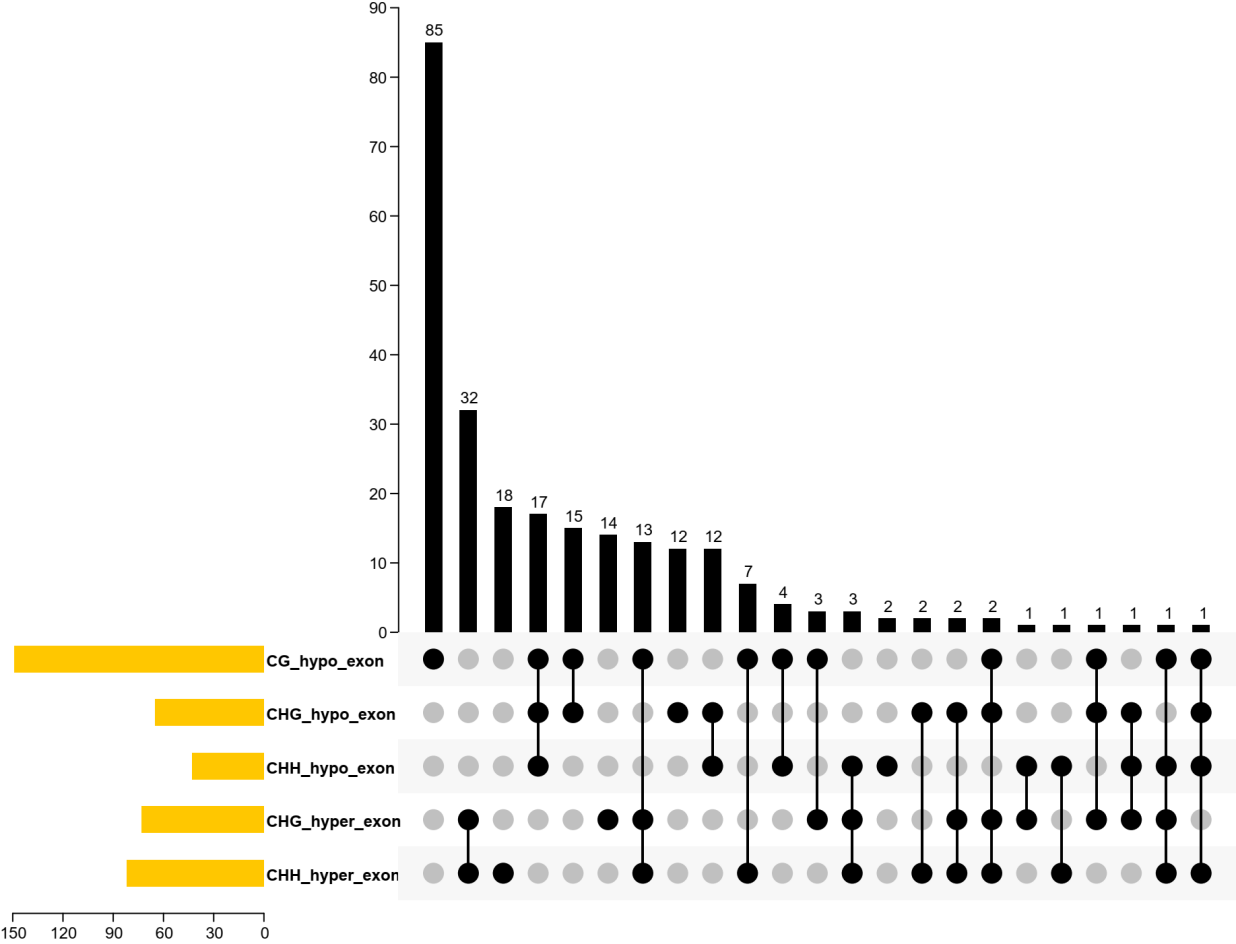


**Figure S17** The overlap of *NB-LRR* genes with 24 h-DMRs at exon region.


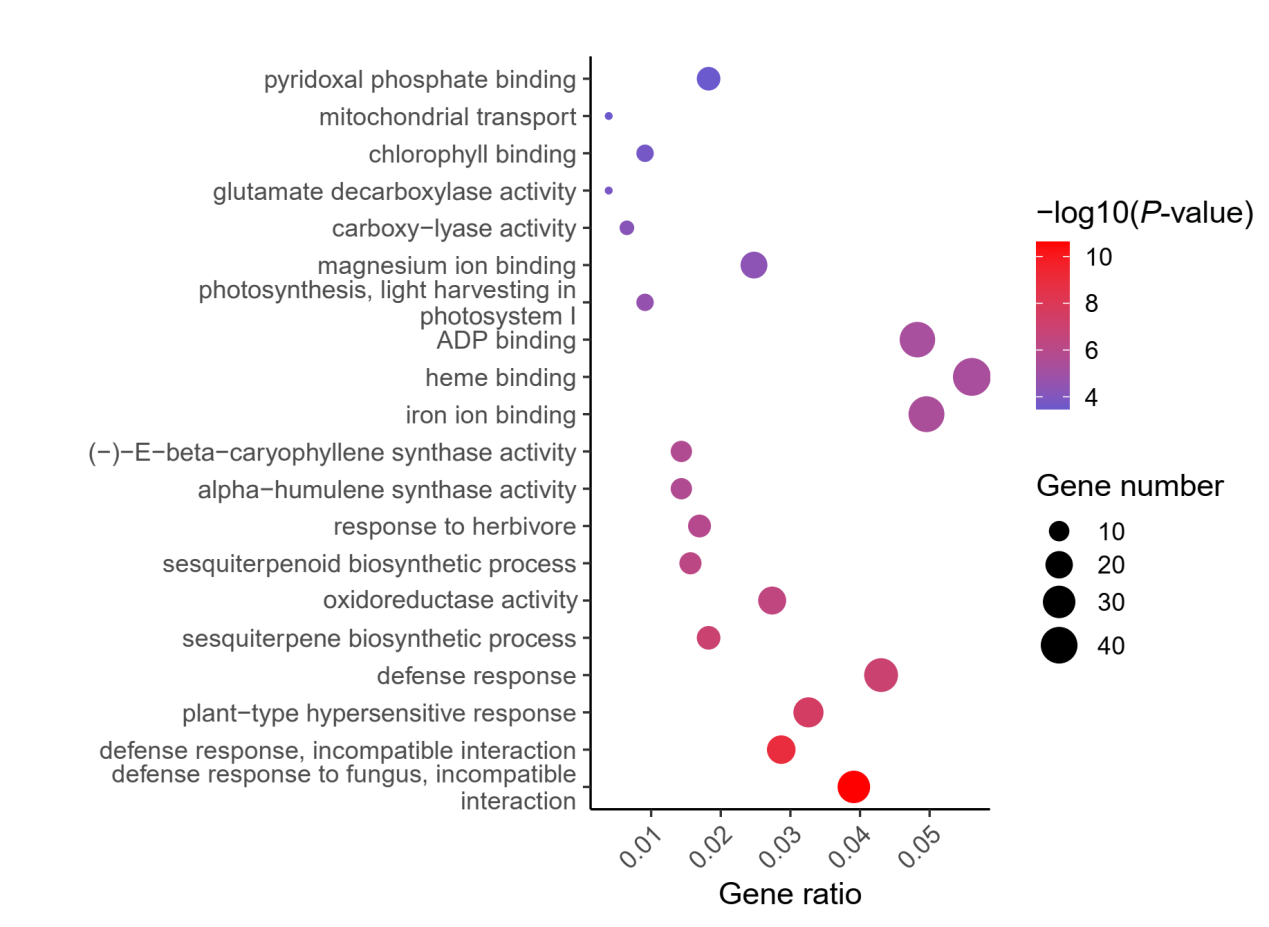


**Figure S18** GO term enrichment of genes with both CHG- and CG-hypo-DMRs at exons.


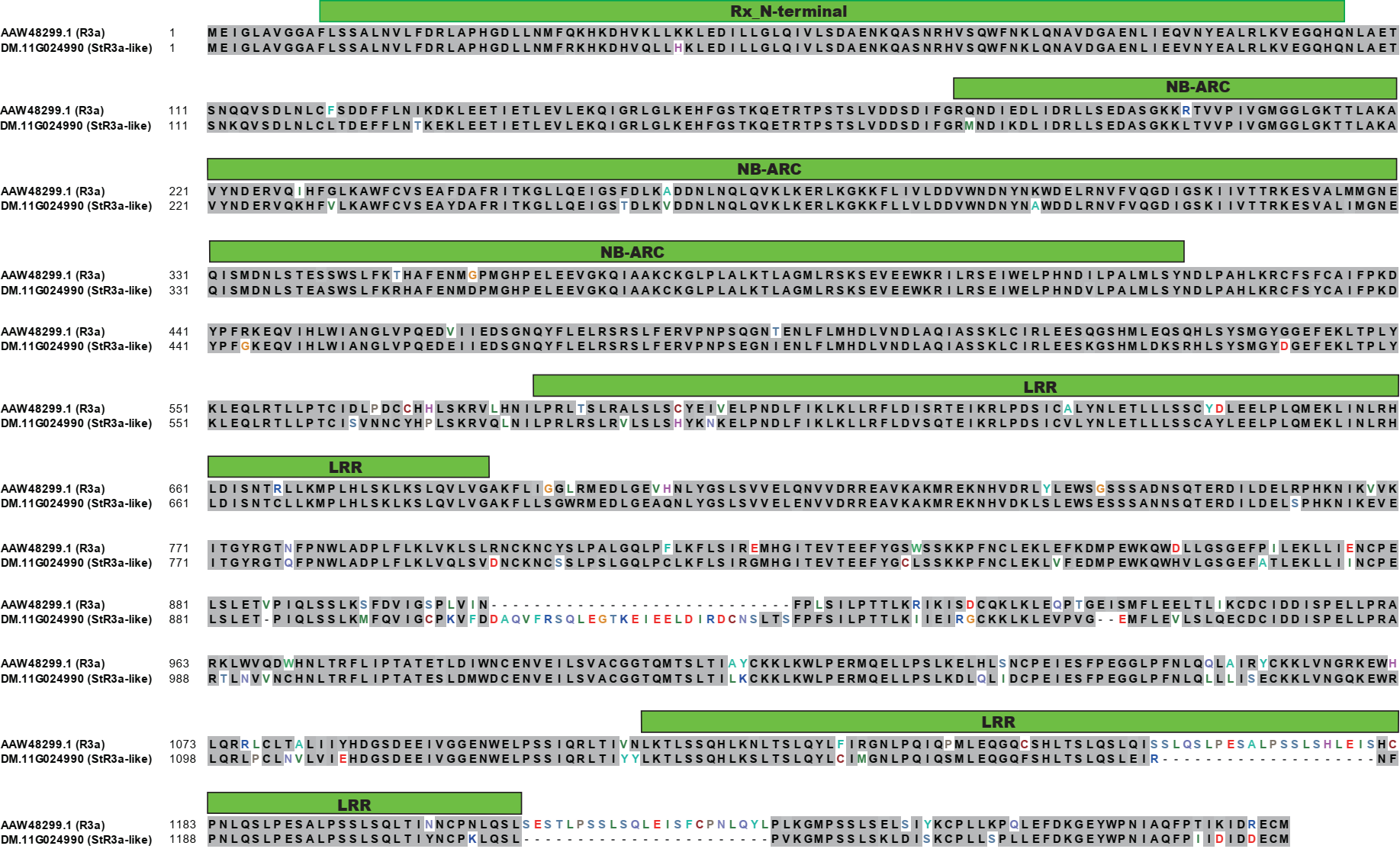


**Figure S19** Comparison of the protein sequence between R3a and R3a-like gene in DM6.


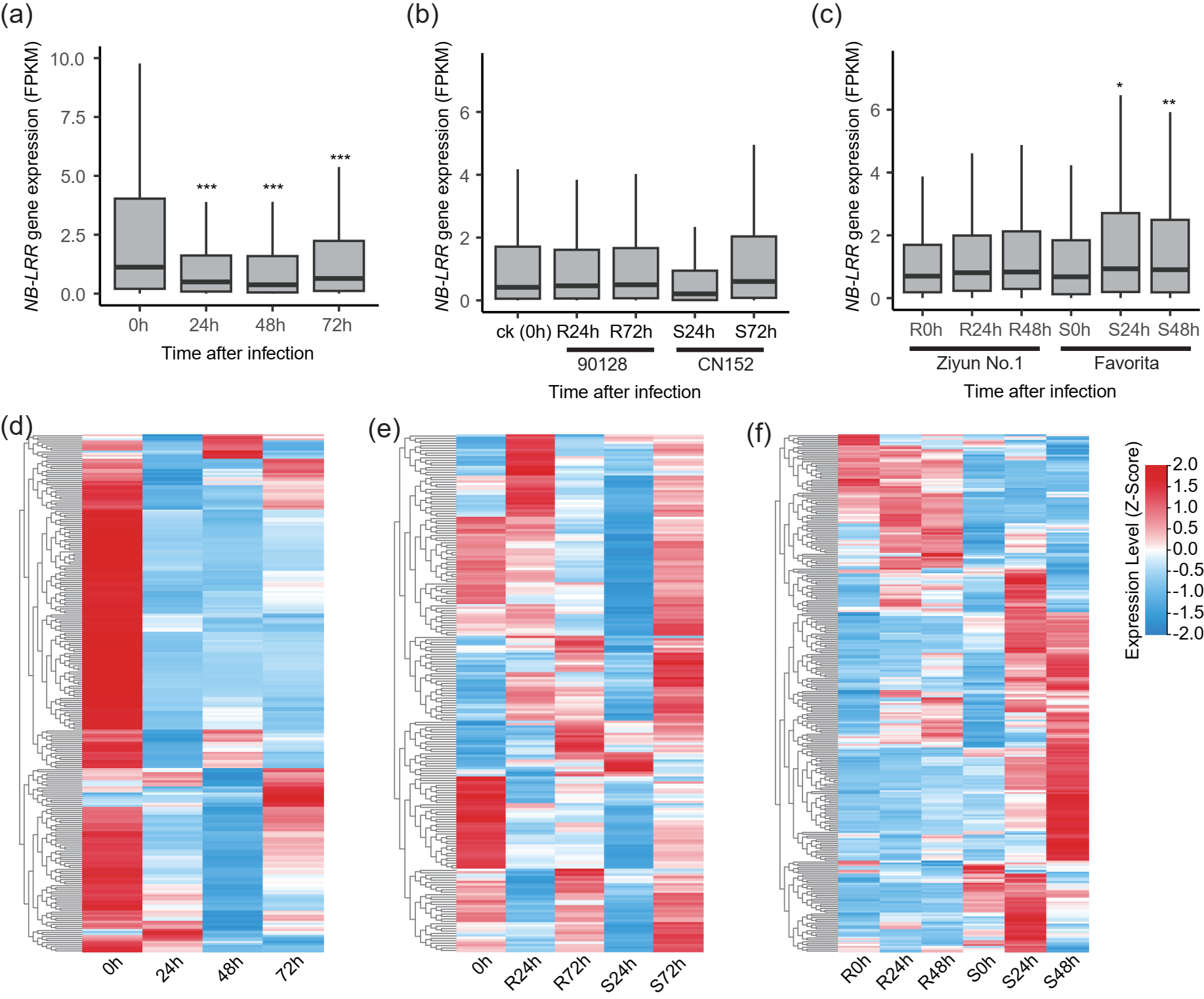


**Figure S20** The expression pattern of *NB-LRRs* in potato’s response to *P. infestans* infection.

(a) Global expression level of *NB-LRRs* in potato QS9 living leaves after *P. infestans* infection. (b) Global expression level of *NB-LRRs* in the resistant or susceptible states of the primitive cultivated potato *S. andigena* inoculated with different strains of *P. infestans*. *S. andigena* shows resistant to strain 90128, but is susceptible to CN152. (c) The resistant and susceptible states of potato cultivar Ziyun No.1 and Favorita to the infection of same *P. infestans* strain, respectively. (d) Heat map show the expression profile of *NB-LRRs* in potato QS9 living leaves after *P. infestans* infection. (e) Heat map show the expression profile of *NB-LRRs* in the resistant or susceptible states of the primitive cultivated potato *S. andigena* inoculated with different strains of *P. infestans*. (f) Heat map show the expression profile of *NB-LRRs* in the resistant and susceptible states of potato cultivar Ziyun No.1 and Favorita to the infection of same *P. infestans* strain, respectively. Only *NB-LRR* with an expression level > 1 FPKM in at least one stage was shown in these heatmaps. “***”, “**”, “*” indicates significant difference between this stage with that of 0h with *P* (two-sample Student’s t-test) less than 0.001, 0.01, 0.05, respectively.


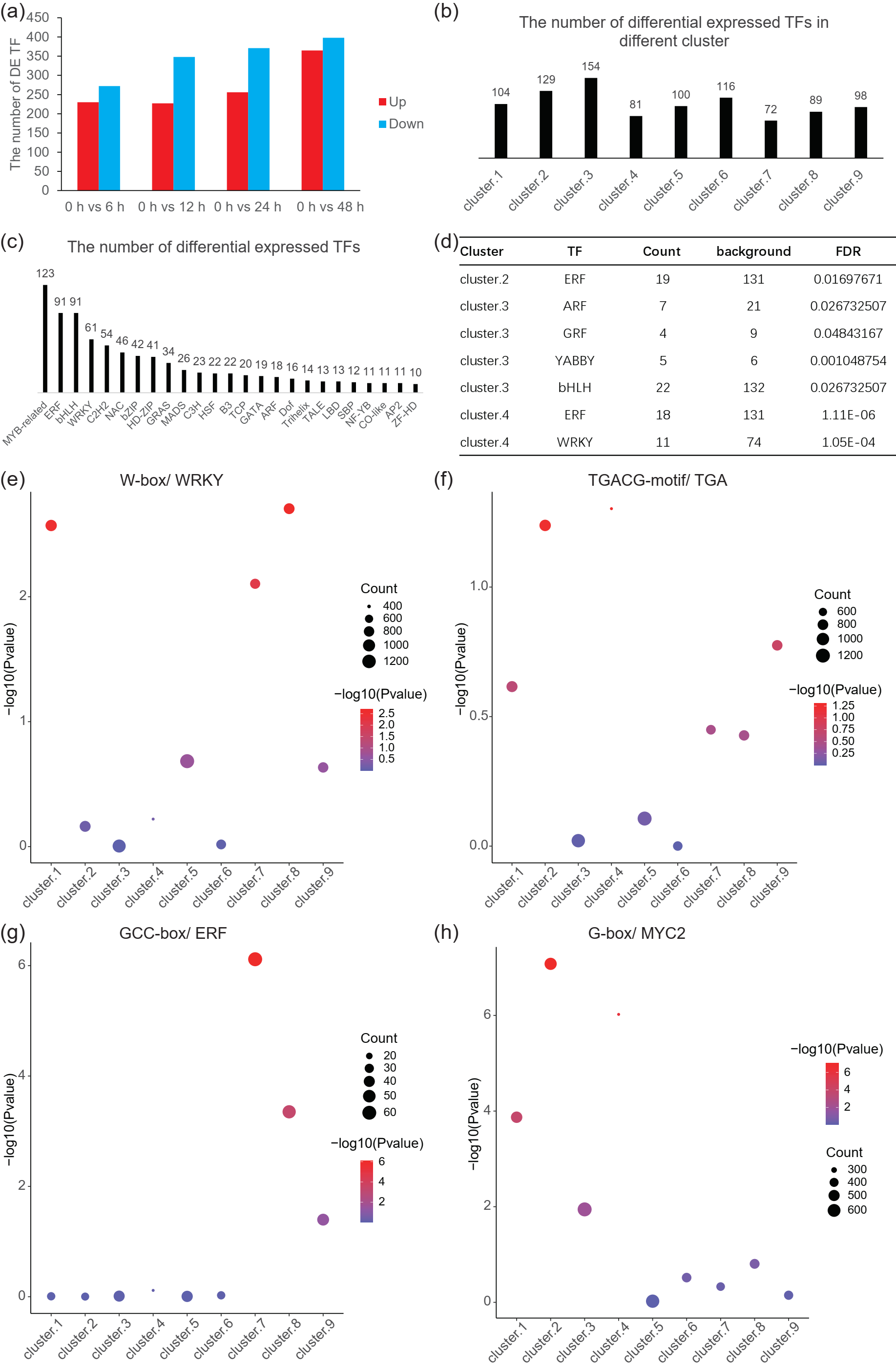


**Figure S21** Potato transcription factors in response to *P. infestans* infection.

(a) The number of differentially expressed TFs in each stage. (b) The number of differentially expressed TFs in each DEG cluster. (c) The number of differentially expressed TFs of different family. (d) The enrichment of differentially expressed TF of different family in each cluster. (e-h) The enrichment of W-box (e), TGACG motif (f), GCC-box (g), and G-box (h) in the promoter of genes from different clusters.


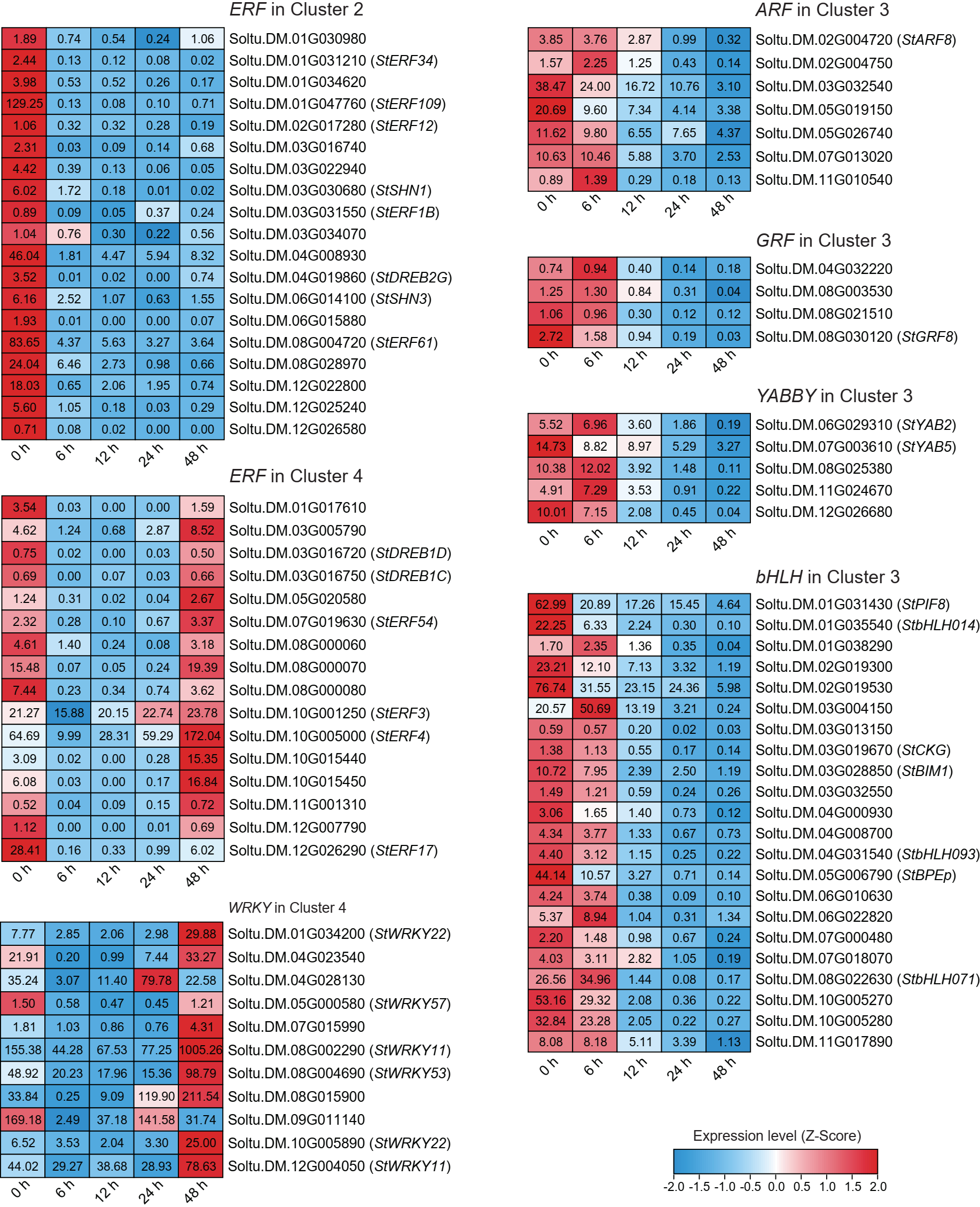


**Figure S22** The expression profile of transcription factors in different clusters.

The values in these squares show the expression level (FPKM).


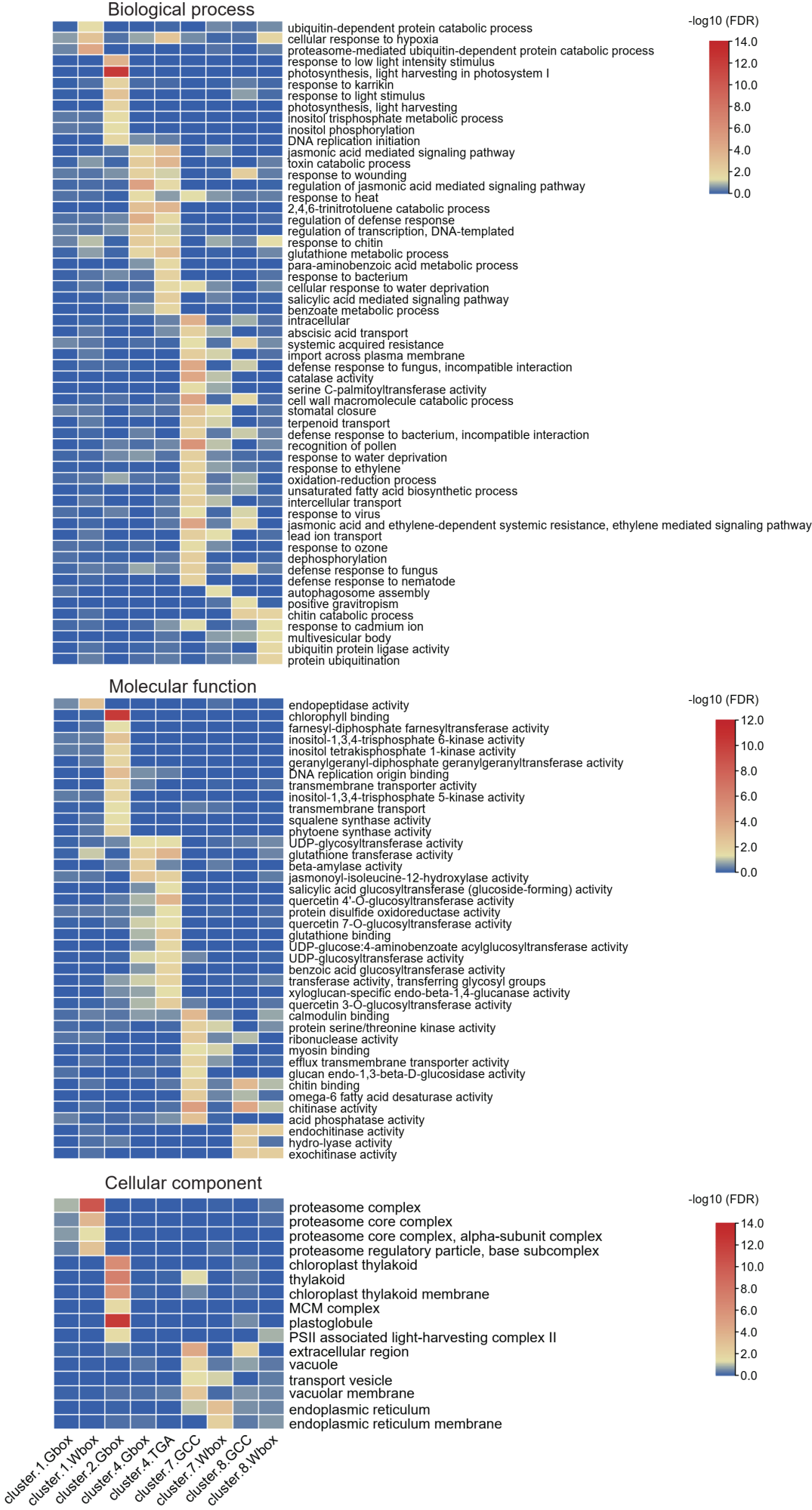


**Figure S23** GO enrichment of genes with enriched *cis*-element in different DEG clusters.

**Table S1** The RNA-seq data and mapping rate in each sample.

| **Sample** | **Raw_bases (G)** | **Raw_reads** | **Clean_reads** | **Total_mapped (%)** | | **Uniquely mapped (%)** | |
| --- | --- | --- | --- | --- | --- | --- | --- |
|  |  |  |  | **Potato** | ***P. infestans*** | **Potato** | ***P. infestans*** |
| 0 h-1 | 20.53 | 68,439,881 | 67,756,877 | 63,847,245 (94.23) | 343,126 (0.51) | 61,401,259 (90.62) | 312,247 (0.46) |
| 0 h-2 | 15.39 | 51,286,773 | 50,709,829 | 47,719,813 (94.10) | 377,428 (0.75) | 45,821,817 (90.36) | 343,879 (0.68) |
| 0 h-3 | 16.99 | 56,629,845 | 55,801,256 | 52,432,560 (93.96) | 521,259 (0.92) | 49,823,919 (89.29) | 473,587 (0.84) |
| 6 h-1 | 10.19 | 33,980,624 | 32,652,930 | 30,383,308 (93.05) | 168,858 (0.50) | 29,464,101 (90.23) | 150,815 (0.45) |
| 6 h-2 | 10.45 | 34,825,887 | 34,175,605 | 31,995,165 (93.62) | 159,260 (0.46) | 30,793,548 (90.10) | 141,093 (0.41) |
| 6 h-3 | 12.33 | 41,099,897 | 39,060,477 | 36,467,637 (93.36) | 165,369 (0.41) | 34,578,918 (88.53) | 146,829 (0.36) |
| 12 h-1 | 11.55 | 38,503,682 | 37,613,683 | 35,193,952 (93.57) | 269,059 (0.70) | 33,463,339 (88.97) | 241,816 (0.63) |
| 12 h-2 | 11.82 | 39,408,450 | 38,570,536 | 34,741,966 (90.07) | 1,265,785 (3.24) | 33,194,392 (86.06) | 1,154,828 (2.96) |
| 12 h-3 | 12.15 | 40,490,814 | 39,037,500 | 36,322,292 (93.04) | 312,530 (0.78) | 34,396,248 (88.11) | 282,374 (0.70) |
| 24 h-1 | 11.59 | 38,633,067 | 37,403,271 | 30,881,055 (82.56) | 3,980,880 (10.41) | 28,748,487 (76.86) | 3,722,513 (9.73) |
| 24 h-2 | 11.15 | 37,151,227 | 36,262,142 | 32,171,017 (88.72) | 1,905,225 (5.17) | 30,476,976 (84.05) | 1,784,024 (4.84) |
| 24 h-3 | 11.34 | 37,806,744 | 37,254,271 | 33,664,139 (90.36) | 1,549,028 (4.13) | 31,415,464 (84.33) | 1,437,556 (3.83) |
| 48 h-1 | 11.96 | 39,867,887 | 39,423,492 | 15,336,060 (38.90) | 21,975,683 (55.62) | 14,220,742 (36.07) | 20,370,815 (51.56) |
| 48 h-2 | 11.31 | 37,709,594 | 37,028,105 | 20,698,198 (55.90) | 13,299,068 (35.64) | 18,251,202 (49.29) | 12,145,390 (32.55) |
| 48 h-3 | 12.49 | 41,622,455 | 40,790,623 | 22,932,916 (56.22) | 15,104,400 (36.61) | 21,730,023 (53.27) | 13,881,412 (33.65) |

**Table S2** WGBS data of potato leaves after *P. infestans* infection.

| Sample | Raw Reads | Clean Reads | Raw Base(G) | Clean Base(G) | Q20  (%) | GC  (%) | BS conversion rate (%) | Uniquely Mapped reads | Uniquely Mapped rate (%) | Coverage |  |
| --- | --- | --- | --- | --- | --- | --- | --- | --- | --- | --- | --- |
| 0 h-1 | 279576914 | 278693852 | 41.94 | 39.02 | 96.91 | 20.82 | 99.69 | 220534341 | 79.13 | 14.64 | |
| 0 h-2 | 273046886 | 272092858 | 40.96 | 38.09 | 96.29 | 21 | 99.69 | 215248344 | 79.11 | 14.32 | |
| 0 h-3 | 326173284 | 324347060 | 48.93 | 45.41 | 96.29 | 20.86 | 99.7 | 256310713 | 79.02 | 16.27 | |
| 6 h-1 | 275130384 | 274636562 | 41.27 | 38.45 | 95.78 | 20.87 | 99.61 | 215137133 | 78.34 | 14.43 | |
| 6 h-2 | 336792004 | 336202584 | 50.52 | 47.07 | 95.81 | 20.58 | 99.62 | 263878434 | 78.49 | 15.92 | |
| 6 h-3 | 271352286 | 270723710 | 40.7 | 37.9 | 96.21 | 21.16 | 99.58 | 211437180 | 78.10 | 14.12 | |
| 12 h-1 | 304712318 | 303931628 | 45.71 | 42.55 | 96.19 | 20.27 | 99.67 | 237670365 | 78.20 | 15.08 | |
| 12 h-2 | 308764706 | 307345966 | 46.31 | 43.03 | 96.27 | 20.61 | 99.67 | 240814821 | 78.35 | 14.69 | |
| 12 h-3 | 272327012 | 270921418 | 40.85 | 37.93 | 95.84 | 20.6 | 99.68 | 212785712 | 78.54 | 13.91 | |
| 24 h-1 | 349214324 | 347277056 | 52.38 | 48.62 | 95.87 | 21.81 | 99.63 | 267516478 | 77.03 | 17.24 | |
| 24 h-2 | 355550726 | 353486442 | 53.33 | 49.49 | 95.81 | 21.72 | 99.66 | 272669384 | 77.14 | 17.28 | |
| 24 h-3 | 331906792 | 329624922 | 49.79 | 46.15 | 96.07 | 21.6 | 99.66 | 253735372 | 76.98 | 16.17 | |

**Table S3** The enrichment of DMRs at non-expressed genes.

| DMR_type | DMG | non_expressed gene overlap (3366) | Fisher's Pvalue |
| --- | --- | --- | --- |
| 6 h.CHH.hyper.Downstream | 65 | 7 | 5.02E-01 |
| 6 h.CHH.hyper.exon | 11 | 1 | 6.95E-01 |
| 6 h.CHH.hyper.intron | 44 | 6 | 2.92E-01 |
| 6 h.CHH.hyper.Upstream | 110 | 11 | 5.78E-01 |
| 6 h.CHH.hypo.Downstream | 26 | 7 | 1.33E-02 |
| 6 h.CHH.hypo.exon | 4 | 1 | 3.50E-01 |
| 6 h.CHH.hypo.intron | 17 | 2 | 5.31E-01 |
| 6 h.CHH.hypo.Upstream | 35 | 2 | 8.86E-01 |
| 12 h.CHH.hyper.Downstream | 6 | 2 | 1.19E-01 |
| 12 h.CHH.hyper.exon | 0 | 0 | 1.00E+00 |
| 12 h.CHH.hyper.intron | 4 | 1 | 3.50E-01 |
| 12 h.CHH.hyper.Upstream | 5 | 0 | 1.00E+00 |
| 12 h.CHH.hypo.Downstream | 247 | 20 | 8.91E-01 |
| 12 h.CHH.hypo.exon | 42 | 4 | 6.35E-01 |
| 12 h.CHH.hypo.intron | 163 | 8 | 9.95E-01 |
| 12 h.CHH.hypo.Upstream | 372 | 25 | 9.93E-01 |
| 24 h.CG.hyper.Downstream | 1994 | 189 | 8.81E-01 |
| 24 h.CG.hyper.exon | 4069 | 243 | 1.00E+00 |
| 24 h.CG.hyper.intron | 2040 | 100 | 1.00E+00 |
| 24 h.CG.hyper.Upstream | 1922 | 160 | 9.98E-01 |
| 24 h.CG.hypo.Downstream | 3653 | 305 | 1.00E+00 |
| 24 h.CG.hypo.exon | 6178 | 341 | 1.00E+00 |
| 24 h.CG.hypo.intron | 3797 | 173 | 1.00E+00 |
| 24 h.CG.hypo.Upstream | 3731 | 328 | 9.99E-01 |
| 24 h.CHG.hyper.Downstream | 2351 | 277 | 6.06E-03 |
| 24 h.CHG.hyper.exon | 3303 | 305 | 9.79E-01 |
| 24 h.CHG.hyper.intron | 3730 | 127 | 1.00E+00 |
| 24 h.CHG.hyper.Upstream | 2188 | 228 | 3.89E-01 |
| 24 h.CHG.hypo.Downstream | 1880 | 245 | 3.64E-05 |
| 24 h.CHG.hypo.exon | 1953 | 348 | 4.52E-26 |
| 24 h.CHG.hypo.intron | 1650 | 128 | 1.00E+00 |
| 24 h.CHG.hypo.Upstream | 2253 | 290 | 1.80E-05 |
| 24 h.CHH.hyper.Downstream | 9160 | 663 | 1.00E+00 |
| 24 h.CHH.hyper.exon | 3615 | 378 | 3.22E-01 |
| 24 h.CHH.hyper.intron | 5375 | 286 | 1.00E+00 |
| 24 h.CHH.hyper.Upstream | 11551 | 716 | 1.00E+00 |
| 24 h.CHH.hypo.Downstream | 1087 | 88 | 9.93E-01 |
| 24 h.CHH.hypo.exon | 1080 | 144 | 5.57E-04 |
| 24 h.CHH.hypo.intron | 1130 | 53 | 1.00E+00 |
| 24 h.CHH.hypo.Upstream | 1566 | 118 | 1.00E+00 |

**Table S4** The public RNA-seq datasets analyzed in this study.

| Title | Potato variety | Sample | Accession | Reference |
| --- | --- | --- | --- | --- |
| Dual RNA Sequencing Reveals the Genome-Wide Expression Profiles During the Compatible and Incompatible Interactions Between *Solanum tuberosum* and *Phytophthora infestans* | Ziyun No.1 | R1 | SRR12357399 | [1] |
|  |  | R2 | SRR12357398 |  |
|  |  | R3 | SRR12357397 |  |
|  | Favorita | S1 | SRR12357396 |  |
|  |  | S2 | SRR12357395 |  |
|  |  | S3 | SRR12357394 |  |
| Comparative Transcriptome Profiling Reveals Compatible and Incompatible Patterns of Potato Toward *Phytophthora infestans* | *Solanum andigena* | ck_1 63 | CRR046156 | [2] |
|  |  | ck_2 59 | CRR046157 |  |
|  |  | ck_3 59 | CRR046158 |  |
|  |  | 90128_24_1 | CRR046159 |  |
|  |  | 90128_24_2 | CRR046160 |  |
|  |  | 90128_24_3 | CRR046161 |  |
|  |  | 90128_72_1 | CRR046162 |  |
|  |  | 90128_72_2 | CRR046163 |  |
|  |  | 90128_72_3 | CRR046164 |  |
|  |  | CN152_24_1 | CRR046165 |  |
|  |  | CN152_24_2 | CRR046166 |  |
|  |  | CN152_24_3 | CRR046167 |  |
|  |  | CN152_72_1 | CRR046168 |  |
|  |  | CN152_72_2 | CRR046169 |  |
|  |  | CN152_72_3 | CRR046170 |  |
| Serial Transcriptome Analysis Reveals Genes Associated with Late Blight Resistance in Potato Cultivar Qingshu 9 | Qingshu No.9 | Pi0h_1 | SRR14695793 | [3] |
|  |  | Pi0h_2 | SRR14695792 |  |
|  |  | Pi0h_3 | SRR14695789 |  |
|  |  | Pi24h_1 | SRR14695788 |  |
|  |  | Pi24h_2 | SRR14695787 |  |
|  |  | Pi24h_3 | SRR14695786 |  |
|  |  | Pi48h_1 | SRR14695785 |  |
|  |  | Pi48h_2 | SRR14695784 |  |
|  |  | Pi48h_3 | SRR14695783 |  |
|  |  | Pi72h_1 | SRR14695782 |  |
|  |  | Pi72h_2 | SRR14695790 |  |
|  |  | Pi72h_3 | SRR14695791 |  |

**Table S5** The primers used for qRT-PCR

| Primer | Sequence (5’-3’) |
| --- | --- |
| PiUBC-F | CATCAATCGGCGTATCTGTCTCA |
| PiUBC-R | CACCAAGTCGGCGAATAGCAC |
| Pi04388-F | GGGCGTGTACGATATGCTCA |
| Pi04388-R | ACAGTCTCTGCGGACTACCT |
| Pi07555-F | AATGAGCCACGCTGCAAAAG |
| Pi07555-R | TCTTCGACCACGTCCAATCC |
| Pi10116-F | GTCGAAGGTCGTGGATTGGA |
| Pi10116-R | CCCGGTATCGAGCTTTAGCA |
| Pi15235-F | AGAAGGTCGGGAGAATTGGC |
| Pi15235-R | AGTGCCCAAGATGCCGAAAA |
| Pi14685-F | ACTCCGTAAATGGGCTGGTG |
| Pi14685-R | TTTCAAGCACCGACTTTGCG |
| Pi13503-F | CAAAGCAGTCGCCAAGAAGC |
| Pi13503-R | CTGCCGGGTGTTTGTTGTTT |
| Pi19302-F | ACAGCGATGATGCTCTGGTC |
| Pi19302-R | GCTTTTGTTCCCGCCTTGTC |
| StEF1α-F | ATTGGAAACGGATATGCTCCA |
| StEF1α-R | TCCTTACCTGAACGCCTGTCA |
| Soltu.DM.04G035830-F | TCCCAAACAGAGGTTGACAGC |
| Soltu.DM.04G035830 -R | CCCGGAGAGCAACTTTCTTGA |
| Soltu.DM.05G006290 -F | AAGCTCCTTCGCCACCTAAG |
| Soltu.DM.05G006290 -R | TCAGACCTCGAAGCAACTGG |
| Soltu.DM.11G018970 -F | TCCAATGCCTGCTTTCCTGT |
| Soltu.DM.11G018970 -R | AGCAGGCAACTTTCCCATCA |

**Reference**

1. Li, H., R. Hu, Z. Fan*, et al.*, Dual RNA Sequencing Reveals the Genome-Wide Expression Profiles During the Compatible and Incompatible Interactions Between *Solanum tuberosum* and *Phytophthora infestans.* *Front Plant Sci*, 2022. **13**: p. 817199.

2. Duan, Y., S. Duan, M.R. Armstrong*, et al.*, Comparative Transcriptome Profiling Reveals Compatible and Incompatible Patterns of Potato Toward *Phytophthora infestans.* *G3-Genes Genom Genet*, 2020. **10**(2): p. 623-634.

3. He, M., Y. Zhou, G. Ye*, et al.*, Serial Transcriptome Analysis Reveals Genes Associated with Late Blight Resistance in Potato Cultivar Qingshu 9*.* *Agronomy*, 2021. **11**(10): p. 1919.
